# Composition and Predominance of *Fusarium* Species Causing *Fusarium* Head Blight in Winter Wheat Grain Depending on Cultivar Susceptibility and Meteorological Factors

**DOI:** 10.3390/microorganisms8040617

**Published:** 2020-04-24

**Authors:** Tim Birr, Mario Hasler, Joseph-Alexander Verreet, Holger Klink

**Affiliations:** 1 Department of Plant Diseases and Crop Protection, Institute of Phytopathology, Christian-Albrechts-University of Kiel, Hermann-Rodewald-Straße 9, 24118 Kiel, Germany; javerreet@phytomed.uni-kiel.de (J.-A.V.); hklink@phytomed.uni-kiel.de (H.K.); 2 Lehrfach Variationsstatistik, Christian-Albrechts-University of Kiel, Hermann-Rodewald-Straße 9, 24118 Kiel, Germany; hasler@email.uni-kiel.de

**Keywords:** *Fusarium* head blight, winter wheat, survey, grain, species composition, DNA amount, deoxynivalenol, zearalenone, cultivar, meteorological factors

## Abstract

*Fusarium* head blight (FHB) is one of the most important diseases of wheat, causing yield losses and mycotoxin contamination of harvested grain. A complex of different toxigenic *Fusarium* species is responsible for FHB and the composition and predominance of species within the FHB complex are determined by meteorological and agronomic factors. In this study, grain of three different susceptible winter wheat cultivars from seven locations in northern Germany were analysed within a five-year survey from 2013 to 2017 by quantifying DNA amounts of different species within the *Fusarium* community as well as deoxynivalenol (DON) and zearalenone (ZEA) concentrations. Several *Fusarium* species co-occur in wheat grain samples in all years and cultivars. *F. graminearum* was the most prevalent species, followed by *F. culmorum*, *F. avenaceum* and *F. poae*, while *F. tricinctum* and *F. langsethiae* played only a subordinate role in the FHB complex in terms of DNA amounts. In all cultivars, a comparable year-specific quantitative occurrence of the six detected species and mycotoxin concentrations were found, but with decreased DNA amounts and mycotoxin concentrations in the more tolerant cultivars, especially in years with higher disease pressure. In all years, similar percentages of DNA amounts of the six species to the total *Fusarium* DNA amount of all detected species were found between the three cultivars for each species, with *F. graminearum* being the most dominant species. Differences in DNA amounts and DON and ZEA concentrations between growing seasons depended mainly on moisture factors during flowering of wheat, while high precipitation and relative humidity were the crucial meteorological factors for infection of wheat grain by *Fusarium*. Highly positive correlations were found between the meteorological variables precipitation and relative humidity and DNA amounts of *F. graminearum*, DON and ZEA concentrations during flowering, whereas the corresponding correlations were much weaker several days before (heading) and after flowering (early and late milk stage).

## 1. Introduction

Fungi of the genus *Fusarium* are known as plurivorous pathogens of various crops worldwide. *Fusarium* diseases are caused by several co-occurring species which infect small-grain cereals like wheat, barley, oat, rye and triticale as well as maize. Many species attack a range of plants parts and stages, such as seedlings, roots, stems and heads, causing *Fusarium* head blight (FHB) of small-grain cereals (also known as scab or ear blight) and ear rot of maize [1,2,3,4].

**Figure 1:**
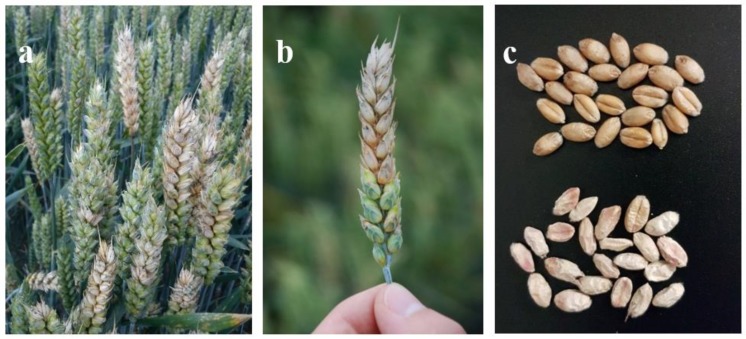
Symptoms of *Fusarium* head blight (FHB) of wheat. (**a**) Symptomatic heads with bleached spikelets. (**b**) Premature bleached head with pinkish-red mycelium and spores on infected spikelets. (**c**) *Fusarium*-damaged grain showing pink and white discolorations (bottom) compared to healthy grain (above).

FHB of wheat (*Triticum aestivum* L.) is one of the most important diseases in wheat-growing regions around the world causing quantitative and qualitative losses. Symptoms of FHB infections usually include premature bleaching of the entire head or just a few spikelets (Figure 1a,b), pinkish-red mycelium and spores on infected spikelets (Figure 1b), inhibited grain formation and grain that are shrivelled, light weighted and discoloured from white to pink as a result of the mycelial outgrowths from the *Fusarium* colonized grain (Figure 1c). If the rachis is infected, the head above the point of infection senesces prematurely due to the undersupply with water and nutrients with corresponding shrivelled, uninfected grain. Together this results in yield losses, reductions of the thousand grain weight (TGW) and germination ability of the harvested grain [1,2,5,6]. Furthermore, the baking quality is adversely affected by the reduction of starch content and degradation of different protein fractions [7]. 

In addition, FHB causes quality losses of grain by the formation of mycotoxins, which causes a potential health risk for animals and humans, because their occurrence in feed and food is often associated with chronic or acute mycotoxicosis. Several *Fusarium* species are capable to produce a range of mycotoxins including zearalenone (ZEA), moniliformin (MON), beauvericin (BEA), enniatins (ENs) and trichothecenes such as deoxynivalenol (DON), nivalenol (NIV), 3- and 15-acetyl-DON (3-AcDON, 15-AcDON), HT-2 and T-2 toxin. The often co-occurring mycotoxins DON and ZEA, mainly produced by the *Fusarium* species *F. graminearum* and *F. culmorum*, are the most common mycotoxins associated with FHB in wheat. Exposure to DON induces chronic effects such as anorexia and reduced growth, as well as acute intoxication leading to vomiting and depressed immune function. ZEA has been proven to be hepatotoxic, immunotoxic and carcinogenic to a number of mammalian species and is able to bind to oestrogen receptors causing reproductive disorders (e.g., hyper-oestrogenism and infertility in livestock) [2,4,8]. Within the EU, limit thresholds have already been defined for DON and ZEA in unprocessed grain and human food based on cereal grain. Limits for unprocessed wheat grain intended for use as food are 1250 µg DON/kg and 100 µg ZEA/kg [9]. Likewise, in other countries of the world, maximum levels for DON and other mycotoxins were established, e.g., Canada, China, Russia and USA [4].

FHB of wheat is caused by a complex of different toxigenic *Fusarium* species. In Europe, the major causal agents of FHB are *F. graminearum*, *F. culmorum*, *F. avenaceum* and *F. poae*. However, *F. equiseti*, *F. langsethiae*, *F. sporotrichioides* and *F. tricinctum* are also frequently found [1,2,10,11,12]. Regional and year-specific differences in the qualitative and quantitative species profile are particularly dependent on meteorological conditions and the cropping system (e.g., crop rotation, previous crop, tillage practices, cultivar susceptibility) [10,13]. Crop residues in the field are the major reservoir on which FHB pathogens saprophytically survive and generally produce asexual conidia, but some also produce sexual ascospores, e.g., *F. graminearum* (teleomorph *Gibberella zeae*) and *F. avenaceum* (teleomorph *Gibberella avenacea*) [2,14]. Conidia are dispersed from crop residues by rain splashing, but wind dispersal is also important, especially for ascospores, which can travel meters to kilometres [15,16,17]. In general, wheat heads are most susceptible to FHB infections at flowering, when the flower is directly exposed to the environment. Warm and moist conditions during this period encourage the development of the disease [1,18,19]. Spores are deposited on or inside wheat florets where they germinate and initiate infection. The fungus infects extruded anthers and then ramifies throughout florets, the developing caryopsis and rachis spreading then to other flowers in the spikelet [20]. Besides flowering, significant precipitation and high relative humidity during the periods of several days before (heading) and after flowering during grain filling increase the occurrence of FHB and mycotoxin contamination [21,22,23,24]. The composition and predominance of *Fusarium* species in wheat is, to a large extent, determined by meteorological factors like moisture (precipitation, relative humidity) and temperature. Traditionally *F. culmorum* and *F. avenaceum* are associated with wet and cool maritime conditions, *F. poae* with relatively drier and warmer conditions, while *F. graminearum* dominates in regions with warm and humid environments [18,19]. Furthermore, FHB infections of wheat are determined by agronomic factors like crop rotation, previous crop, and soil cultivation. FHB epidemics are favoured when wheat follows host crops including small-grain cereals and especially silage maize and grain maize together with reduced cultivation tillage systems that preserve organic residues from the previous crop on the soil surface, suitable for saprophytic survival of the fungi and serving as a source of inoculum for *Fusarium* spores [14,20,25,26,27]. Different wheat cultivars are known to express different levels of susceptibility towards FHB and therefore, the choice of cultivar is currently the most effective agronomic method to reduce FHB infections and mycotoxin contamination [27,28,29].

Due to differences in pathogenicity, toxigenicity, and fungicide sensitivity, it is important to obtain detailed knowledge of the qualitative and quantitative occurrence of individual *Fusarium* species [11]. The visual assessment of disease severities and incidences of affected heads, spikelets or damaged grain at harvest only estimate disease symptoms, but not pathogen populations. In contrast, mycological methods based on the stimulation of fungal growth from grain (or heads) infected by *Fusarium* on agar plates and subsequent morphological analysis determine the spectrum and the incidence of the different FHB-causing species. The standard parameter is therefore the percentage of infected grain by the different FHB-causing fungi. These methods require taxonomical expertise, are time expensive and risk the overestimating of fast-growing species [30]. Compared to the aforementioned methods, polymerase chain reaction (PCR)-based methods using species-specific primers have the advantage of species specificity, sensitivity and speed without the need for isolation and cultivation of the fungi from grain or heads. Furthermore, quantitative PCR (qPCR) offers the opportunity for quantifying DNA amounts and reveals the true dominance of the individual species in the FHB complex [11,31].

In the present study, harvested winter wheat grain of different susceptible cultivars from maize-free crop rotations was collected in a five-year survey from 2013 to 2017 at seven locations in northern Germany, a suitable growing area for wheat. Besides the determination of mycotoxin contamination, the *Fusarium* community in wheat grain was characterized by species-specific qPCR assays. The aims of the study were (i) to characterize the year-specific composition and quantitative predominance of species belonging to the genus *Fusarium* by quantifying DNA amounts as well as DON and ZEA concentrations, (ii) to analyse interactions between the occurring species, (iii) to determine differences in the qualitative and quantitative species complex and mycotoxin concentrations between different susceptible wheat cultivars, and (iv) to evaluate the effects of regional meteorological factors before, during and after flowering on DNA amounts of the different species in the *Fusarium* community and DON and ZEA concentrations in order to characterize the most crucial stages of wheat development towards FHB infections and mycotoxin contamination.

## 2. Materials and Methods

### 2.1. Area Surveyed and Survey Strategy

The northernmost federal state of Germany, Schleswig-Holstein, was used as an exemplary study area. In the five-year survey from 2013 to 2017, grain samples of winter wheat were annually analysed for the qualitative and quantitative occurrence of different *Fusarium* species and DON and ZEA concentrations at seven trial locations. The region between the North and Baltic Sea is characterized by maritime weather conditions, with an average annual temperature of 8.9 °C and an annual precipitation of 823 L/m^2^ [32] and can be further divided into three areas according to soil type and resulting land use. The western (West Coast Marsh) and eastern parts (Eastern Hill Land) are characterized by heavy soils and large acreages of winter wheat followed by oilseed rape, silage maize and winter barley, while the midland between those regions is dominated by sandy soils and high coverage of grassland and silage maize. Arable crops were grown on 651,000 ha in 2017 with winter wheat as the dominant crop in crop rotation (28.9% of arable land) followed by silage maize (24.7%), oilseed rape (14.9%) and winter barley (10.3%) [33]. The seven trial locations were located in the two main producing areas for winter wheat (West Coast Marsh, Eastern Hill Land) as part of a regional monitoring for leaf pathogens (IPM wheat model) [34] and FHB in wheat [35] (Table 1). During the five-year survey, winter wheat and winter oilseed rape preceded wheat in 57% and 43% of the monitoring locations, respectively (Table 1). Soil cultivation by ploughing was the most commonly used cultivation practice (57%) followed by reduced cultivation tillage (43%), which was only applied after oilseed rape. Soil cultivation and previous crop were the same across all years at a location, as shown in Table 1. Mineral fertilizer application averaged 220 to 240 kg N/ha.

In all years of our five-year survey and from all locations, grain samples of three cultivars with different susceptibility levels towards FHB infections (highly, moderately to highly and lowly to moderately susceptible) were analysed in order to determine differences in the composition and quantitative predominance of the occurring *Fusarium* species as well as DON and ZEA concentrations depending on cultivar susceptibility. These different susceptible levels were represented by the cultivars “Ritmo” (highly susceptible; susceptible category 7), “Inspiration” (moderately to highly susceptible; susceptible category 6) and “Dekan” (lowly to moderately susceptible; susceptible category 4). The susceptibility of wheat cultivars to FHB is scaled into nine susceptibility categories from 1 (missing to very low) to 9 (very high) by the Federal Plant Variety Office [36], an independent senior federal authority under the supervision of the Federal Ministry of Food and Agriculture of Germany. In the following, only the susceptibility levels of the cultivars (highly, moderately to highly and lowly to moderately susceptible) are mentioned and not the cultivar itself. Grain samples were collected from three control plots (= three replications) per cultivar and sampling site, without fungicide treatments or artificial *Fusarium* inoculations, which were integrated in farmers’ fields [35]. Each field plot with a size of 10 m^2^ per plot (2 × 5 m) was harvested with a plot combine and a sample of 1000 g grain per plot was taken for DNA and mycotoxin extraction. Crop management and harvesting were carried out in cooperation with the Chamber of Agriculture of Schleswig-Holstein.

### 2.2. Meteorological Data

Precipitation (mm = L/m^2^; measuring accuracy ± 3%), relative humidity (%; measuring accuracy ± 2%) and air temperature (°C; measuring accuracy ± 0.1 K) were recorded at 30 cm above the ground with meteorological stations (Thies Clima, Göttingen, Germany), which were located directly in the field at each monitoring location. The data were recorded in 15 s intervals and were automatically given as hourly values. Daily cumulative precipitation was estimated from hourly precipitation values; daily means of relative humidity and temperature were averaged from hourly relative humidity and temperature values, respectively. Cumulative precipitation (PRE), mean relative humidity (RH) and mean temperature (TEMP) were calculated for different time periods of wheat development by adding up all daily cumulative precipitation and averaging all daily means of relative humidity and temperature. These different periods of wheat development with a respective duration of 7 days were: (1) 10 to 4 days before growth stage (GS) 65 (corresponding to heading = GS 51 to 59), (2) 7 days around GS 65 (± 3 days around mean flowering date; corresponding to flowering period = GS 61 to 69), (3) 4 to 10 days after GS 65 (corresponding to early milk stage = GS 73) and (4) 11 to 18 days after GS 65 (corresponding to late milk stage = GS 77). Growth stages were determined in weekly intervals from GS 30 to 83 as part of a regional monitoring for leaf pathogens (IPM wheat model) by visual assessment according to Zadoks et al. [37] at each location and year. Additionally, the period of flowering was examined to determine the mean flowering date (GS 65 = flowering halfway = anthers occurring halfway to tip and base of the head) for calculating the abovementioned periods. Therefore, ten main tillers per plot were labelled diagonally through the plot and were analysed daily by noting the presence of emerged anthers according to Zadoks et al. [37] by employees of the chamber of Agriculture of Schleswig-Holstein.

### 2.3. Sample Preparation

Wheat grain samples were ground in a mill (Fritsch, Idar-Oberstein, Germany) to 0.2 mm particle size for DNA and mycotoxin extraction. If necessary, grain samples were dried to a standard water content of 14% after harvest.

### 2.4. Fungal Isolates

The fungal isolates used for quantitative PCR (qPCR) were obtained from the DSMZ (Braunschweig, Germany). The extracted DNA from each isolate was used for positive controls and standard curves for the determination of DNA amounts and primer efficiencies. The following isolates were used: *F. graminearum* (DSM-1095), *F. culmorum* (DSM-1094), *F. poae* (DSM-62376), *F. avenaceum* (DSM-21724), *F. equiseti* (DSM-21725), *F. sporotrichioides* (DSM-62425) and *F. tricinctum* (DSM-21783). *F. langsethiae* (*F. langsethiae* 8051) was obtained from Aarhus University, Faculty of Agricultural Sciences, Department of Integrated Pest Management, Denmark. The *Fusarium* isolates were grown on potato dextrose agar (PDA; Carl Roth, Karlsruhe, Germany) for 2 weeks at 20 °C under 12 h light and 12 h darkness.

### 2.5. DNA Extraction

DNA was extracted from 150 mg ground grain material of each field plot for each of the three cultivars and seven sampling sites using the NucleoSpin^®^Plant II extraction kit (Macherey-Nagel, Düren, Germany) according to the manufacturer’s description. The mycelium of each fungal isolate was scraped off the PDA and ground in liquid N_2_. DNA was extracted from homogenized mycelium using the DNeasy extraction kit (Qiagen, Hilden, Germany) according to the manufacturer’s instructions. The concentration and purity of DNA from the fungal isolates used for standard curves and from the extracted DNA from grain samples was determined using a NanoDropTM OneC (Thermo Scientific, Waltham, MA, USA). DNA concentrations of wheat grain samples were adjusted to 20 ng/µL. DNA samples were stored at −20 °C.

### 2.6. Quantitative PCR (qPCR)

The identification and quantification of eight different *Fusarium* species in wheat grain samples was carried out by qPCR using a qTOWER 2.2 (Analytik Jena, Jena, Germany). qPCR was performed on DNA isolated from wheat grain samples with species-specific primers designed by Nicolaisen et al. [31] for the *Fusarium* species *F. graminearum*, *F. culmorum*, *F. poae*, *F. avenaceum*, *F. equiseti*, *F. sporotrichioides*, *F. tricinctum* and *F. langsethiae* and for plant DNA. The qPCR assays had a total volume of 20 µL, with 10 µL SsoAdvanced^TM^SYBR^®^-Green Super-mix (Bio-Rad Laboratories, Hercules, CA, USA), 2 µL total genomic DNA (20 ng/µL), 6 µL water (HPLC grade) and 1 µL of each primer (10 pmol/µL). PCR reactions were carried out in triplicate for each sample. The following cycling protocol was used for each *Fusarium* species primer assay: 2 min at 50 °C; 95 °C for 10 min; 40 cycles of 95 °C for 15 s and 62 °C for 1 min followed by dissociation analysis from 60–95 °C. For the plant assay, annealing and extension were performed at 60 °C [31]. Wheat samples were analysed using the different *Fusarium* primer assays together with the plant assay as a positive control. A standard curve for each *Fusarium* and plant assay was run with pure fungal and wheat DNA in a five-fold dilution series (0, 0.05, 0.5, 5, 50 ng/µL) using the aforementioned *Fusarium* isolates and healthy wheat grain for plant DNA. The results of each individual field sample from each species-specific and plant assay were evaluated by studying the dissociation curve and cycle threshold (Ct) value. The amount of fungal DNA was calculated from the Ct values using the standard curve, and these values were normalized with the estimated amount of plant DNA based on the plant assay. The DNA amount was calculated as pg fungal DNA per ng plant DNA according to Nicolaisen et al. [31]. For the determination of primer efficiencies, the standard curve for each of the primer assays was used and the efficiency (E = 10^(−1/slope)^) was calculated from the slope of the linear relationship of the log_10_ values of the DNA concentration and the cycle number (Ct) [11]. Standards were included in every plate to estimate E values and to account for differences in qPCR efficiencies between runs.

### 2.7. Analysis of Mycotoxins

The mycotoxins DON and ZEA were extracted separately from milled grain samples (10 g per plot and mycotoxin) and were cleaned up according to Birr et al. [35]. For clean-up of DON and ZEA, the DONPREP^®^ column (R-Biopharm Rhone, Glasgow, UK) and MycoSep^®^226 AflaZon+ column (Romer Labs^®^, Union, MO, USA) were used, respectively. The identification and quantification of DON and ZEA was performed by LC/MS on a 500-MS LC/MS-MS ion trap/ESI system (Varian, Palo Alto, CA, USA) with a reversed-phase Polaris 5 C18-A, 150 × 3 mm column with a particle size of 5 μm (Agilent Technologies, Santa Clara, CA, USA) as described by Birr et al. [35].

### 2.8. Statistical Analysis

The statistical software R, version 3.5.3 (R Foundation for Statistical Computing, Vienna, Austria), was used to evaluate the data using the packages multcomp [38] and nlme [39]. The data evaluation started with the definition of an appropriate statistical mixed model [40,41]. The data were assumed to be approximately normally distributed and to be heteroscedastic due to the different levels of *Fusarium* species and cultivar. These assumptions are based on a graphical residual analysis. The statistical model included the *Fusarium* species (*F. graminearum*, *F. culmorum*, *F. poae*, *F. avenaceum*, *F. equiseti*, *F. sporotrichioides*, *F. tricinctum* and *F. langsethiae*), cultivar (highly, moderately to highly and lowly to moderately susceptible) and year (2013, 2014, 2015, 2016, 2017), as well as their interaction terms as fixed factors. Replications, nested in cultivar and nested in location, were regarded as random factors. The correlations of DNA amounts due to the several levels of species were also taken into account. Based on this model, a Pseudo *R^2^* was calculated [42] and an analysis of variances (ANOVA) was conducted to determine the effect of the fixed factors year, cultivar and species and their interactions on DNA amounts (factor/interaction significance *p* ≤ 0.05), followed by corresponding multiple contrast tests [43,44] in order to compare the several levels of species, years and cultivars, respectively. Mean values labelled with the same letter are not significantly different from each other (*p* > 0.05). The same model was used to analyse the effect of year and cultivar on DON and ZEA concentrations. The model was equal to the first except that DNA amounts were replaced by DON or ZEA concentrations and species as a fixed factor was eliminated as a redundant part of the model. 

Pearson correlation coefficients were calculated to determine the degree of linear relationship between DNA amounts of the different detected *Fusarium* species and meteorological variables (PRE, RH, TEMP) for the three different susceptible cultivars and four different periods of wheat development (heading, flowering, early and late milk stage). These analyses were also performed for DON and ZEA concentrations and meteorological variables. Pearson correlation analysis was also used to evaluate correlations between DNA amounts of DON and ZEA producers *F. graminearum* and *F. culmorum* and concentrations of DON and ZEA. 

## 3. Results

### 3.1. Meteorological Conditions

**Figure 2:**
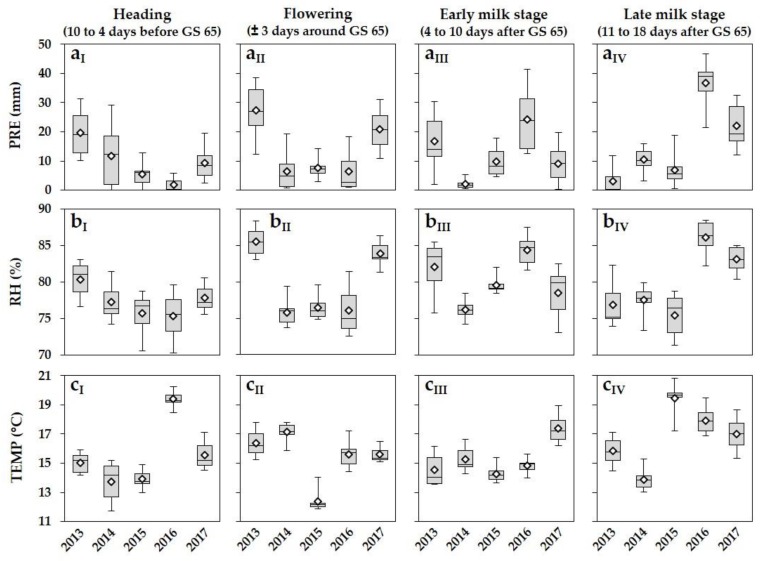
Boxplots and means (white rhombus) of meteorological variables (**a**) PRE (cumulative precipitation, mm), (**b**) RH (relative humidity, %) and (**c**) TEMP (mean temperature, °C) 10 to 4 days before GS 65 (heading; I), ± 3 days around GS 65 (flowering; II), 4 to 10 days after GS 65 (early milk stage; III) and 11 to 18 days after GS 65 (late milk stage; IV) of wheat of the seven trial locations in northern Germany from 2013 to 2017. Five statistics are represented in each boxplot from bottom to top: the smallest observation, lower quartile, median, upper quartile, and largest observation, respectively.

Within the five-year survey from 2013 to 2017, the meteorological variables PRE, RH and TEMP varied substantially between years and locations within a year during the periods 10 to 4 days before GS 65 (heading), ± 3 days around GS 65 (flowering), 4 to 10 days after GS 65 (early milk stage) and 11 to 18 days after GS 65 (late milk stage) (Figure 2).

Ten to four days before GS 65 (heading), the PRE varied considerably between years. The highest mean values were measured in 2013 with 19.6 mm followed by 2014 (11.7 mm) and 2017 (9.2 mm), whereas only low PRE values were recorded in 2015 and 2016 (Figure 2a_I_). The RH values reached a similar level between 2014 and 2017 (75.3% to 77.0%) and were higher in 2013 with 80.6% (Figure 2b_I_). High fluctuations of PRE and RH were observed between locations within a year. Higher mean TEMP was present in 2016 (19.4 °C) followed by the remaining years with lower mean values from 13.8 to 15.5 °C (Figure 2c_I_).

The years 2014, 2015 and 2016 were characterized by the lowest mean values in the meteorological variables PRE (2014: 6.5 mm, 2015: 7.5 mm, 2016: 6.4 mm) and RH (2014: 75.8%, 2015: 76.4%, 2016: 76.1%) during the period ± 3 days around GS 65 (flowering) (Figure 2a_II_,b_II_). In contrast, the flowering period in 2013 and 2017 remained wetter with significantly higher mean values of PRE (2013: 27.3 mm, 2017: 20.8 mm), which resulted in a higher RH of 85.5% in 2013 and 83.9% in 2017, respectively. In all years PRE and RH fluctuated considerably between locations within a year. The TEMP was comparable in 2013, 2014, 2016 and 2017, with mean values from 15.6 to 17.1 °C, whereas lower values were recorded in 2015 with a mean of 12.4 °C (Figure 2c_II_).

In the period 4 to 10 days after GS 65 (early milk stage), the highest mean values of PRE and RH were determined in 2013 and especially in 2016 (Figure 2a_III_,b_III_), a year with lower values of the aforementioned meteorological variables during flowering. PRE and RH varied substantially between locations within each year. The mean TEMP reached a similar level from 2013 to 2016, whereas higher TEMP was recorded in 2017 (Figure 2c_III_).

During the late milk stage (11 to 18 days after GS 65), much higher PRE and RH values were detected in 2016 (PRE = 36.6 mm; RH = 86.1%) and 2017 (PRE = 22.2 mm; RH = 83.1%) compared to the previous years (Figure 2a_IV_,b_IV_).

### 3.2. Fusarium Species in Wheat Grain

Six *Fusarium* species associated with FHB were recurrently detected in wheat grain samples of the highly susceptible cultivar within the five-year survey (Table 2). The most frequent species were *F. graminearum*, *F. culmorum*, *F. avenaceum* and *F. poae*, which were found in ≥ 80% of all samples through the period of study and in all samples in 2013 and 2017. Lower frequencies were observed in 2014, 2015 and 2016. A high percentage of infected grain samples were also detected for *F. tricinctum* and *F. langsethiae* across years with 67% and 65%, respectively. Regardless of cultivar susceptibility, the same *Fusarium* species complex was found in the moderately to highly and the lowly to moderately susceptible cultivar (Tables S1 and S2). The percentages of infected grain samples were comparable between these three different susceptible cultivars in all years.

### 3.3. Effect of Year and Cultivar Susceptibility on Fusarium DNA Amounts, DON and ZEA Concentrations

Averaged over all years, cultivars and detected *Fusarium* species, ANOVA results showed that DNA amounts in wheat grain samples were significantly affected by the interaction of year, cultivar and species (Table 3). Comparative to this, no significant interaction was found for year and cultivar averaged over all detected species, indicating that the influence of this interaction differs from species to species as shown before for the significant triple interaction of year, cultivar and species. Significant two-fold interactions on DNA amounts were determined for year and species, and cultivar and species. All single factors had a significant effect. DON and ZEA concentrations were significantly affected by year and cultivar and its interaction.

As shown in Table 4, positive correlations of DNA amounts were found between *F. culmorum* and *F. avenaceum*, *F. graminearum* and *F. culmorum*, and between *F. poae* and *F. tricinctum*. All other species combinations were only weakly or not correlated.

**Figure 3:**
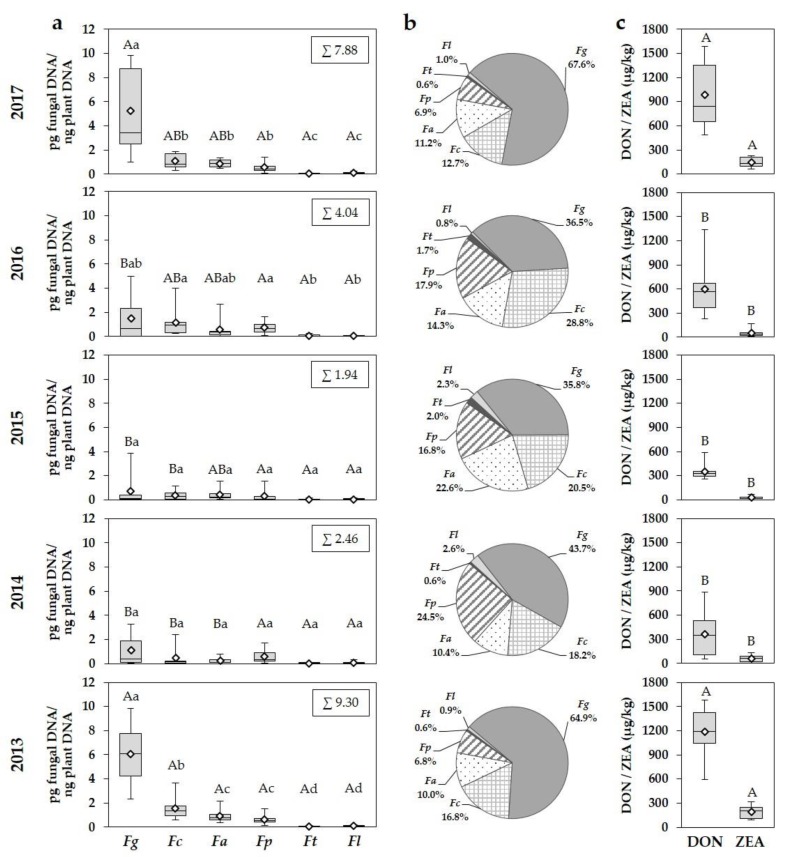
Boxplots and means (white rhombus) of (**a**) DNA amounts of *F. graminearum* (*Fg*), *F. culmorum* (*Fc*), *F. avenaceum* (*Fa*), *F. poae* (*Fp*), *F. tricinctum* (*Ft*) and *F. langsethiae* (*Fl*) (pg fungal DNA/ng plant DNA), (**b**) percentages of DNA amounts of the detected *Fusarium* species to the total *Fusarium* DNA amount of all detected species and boxplots and means (white rhombus) of (**c**) DON and ZEA concentrations (µg/kg) in wheat grain of the highly susceptible cultivar of the seven trial locations (three replications per location) in northern Germany from 2013 to 2017. Five statistics are represented in each boxplot from bottom to top: the smallest observation, lower quartile, median, upper quartile, and largest observation, respectively. Mean values labelled with the same letter (small or big) are not significantly different from each other (*p* > 0.05). Capital letters describe differences for one species and DON and ZEA concentrations between years and small letters differences between the detected *Fusarium* species within a year. *n* = 105.

Exemplified for the highly susceptible cultivar, DNA amounts of *F. graminearum*, *F. culmorum*, *F. avenaceum*, *F. poae*, *F. tricinctum* and *F. langsethiae* in wheat grain differed considerably between years and between species within a year (Figure 3a). During the five-year survey the highest DNA amounts were detected in 2013 and 2017. These years were characterized by the highest values of PRE and RH during flowering (Figure 2a_II_,b_II_). In 2014, 2015 and 2016, significantly lower DNA amounts were analysed (Figure 3a).

In all years, *F. graminearum* occurred as the most dominant species in the FHB complex. Especially in 2013 and 2017, significantly higher DNA amounts were analysed with 6.04 and 5.25 pg fungal DNA/ng plant DNA compared to 2014, 2015 and 2016 with 1.08, 0.69 und 1.48 pg fungal DNA/ng plant DNA (Figure 3a). Compared to all other species, significantly higher DNA amounts were observed in 2013 and 2017 (Figure 3a), resulting in the highest percentages of the total *Fusarium* DNA of all detected *Fusarium* species with 64.9% and 67.6% (Figure 3b). Additionally, in years with lower infection pressure, DNA amounts of *F. graminearum* reached the highest percentages of the total *Fusarium* DNA with 43.7% in 2014, 35.8% in 2015 and 36.5% in 2016, respectively. In all years, DNA amounts of *F. graminearum* fluctuated considerably between locations within a year (Figure 3a), attributable to the varying meteorological conditions during flowering (Figure 2a_II_,b_II_).

Compared to *F. graminearum*, all other species appeared with lower DNA amounts, whereby *F. culmorum*, *F. avenaceum* and *F. poae* were the most important species in the FHB complex in terms of DNA amounts (Figure 3a). Higher DNA amounts of *F. culmorum* were found in 2013, 2016 and 2017 with 1.57, 1.64 and 1.08 pg fungal DNA/ng plant DNA, whereas lower DNA amounts were detectable in 2014 and 2015. Nevertheless, *F. culmorum* gained a greater importance in the FHB complex in terms of DNA amounts in almost all years with percentages of the total *Fusarium* DNA from 12.7% to 28.8%, respectively. The highest DNA amounts of *F. avenaceum* were detected in 2013 and 2017 with 0.93 and 0.88 pg fungal DNA/ng plant DNA compared to the remaining years. *F. avenaceum* reached percentages of the total *Fusarium* DNA from 10.0% to 22.6%. *F. poae* occurred with similar DNA amounts in all years and reached higher percentages of the total *Fusarium* DNA in years with lower disease pressure (2014: 24.5%, 2015: 16.8%, 2016: 17.9%). With the exception of 2013, no significant differences in DNA amounts were found between *F. culmorum*, *F. avenaceum* and *F. poae*. *F. tricinctum* and *F. langsethiae* played only a subordinate role in the FHB complex in all years with maximum percentages of the total *Fusarium* DNA of 2.0% and 2.6%, respectively (Figure 3a,b).

According to the increased occurrence of the DON and ZEA producers *F. graminearum* and *F. culmorum* in 2013 and 2017, the highest DON concentrations were also observed in these years with a mean of 1186 and 987 μg/kg, respectively (Figure 3c). Significantly lower concentrations were detected in 2014, 2015 and 2016. The European maximum level of 1250 µg DON/kg for unprocessed wheat grain intended for use as food was exceeded in 20% of all grain samples within the five-year survey (2013: 48%; 2014, 2015: 0%; 2016: 14%; 2017: 38%). Similar to DON, significantly higher ZEA concentrations were also found in 2013 and 2017 with a mean of 189 and 146 μg/kg compared to 2014, 2015 and 2016. From 2013 to 2017, 31% of all samples contained more than the European maximum level of 100 µg ZEA/kg (2013: 71%; 2014: 14%; 2015: 0%; 2016: 14%; 2017: 57%). According to the fluctuating DNA amounts of *F. graminearum* and *F. culmorum*, DON and ZEA concentrations also showed a large variation between locations within a year.

As shown for the highly susceptible cultivar (Figure 3), a comparable year-specific quantitative occurrence of the six detected *Fusarium* species as well as DON and ZEA concentrations were found in the moderately to highly and the lowly to moderately susceptible cultivar, but with a reduced expression of DNA amounts and decreased DON and ZEA concentrations in the more tolerant cultivars (Figures S1 and S2). In all years, comparable percentages of DNA amounts of the six detected species to the total *Fusarium* DNA were found for each species between the three different susceptible cultivars (Figure 3b, Figures S1b and S2b).

**Figure 4:**
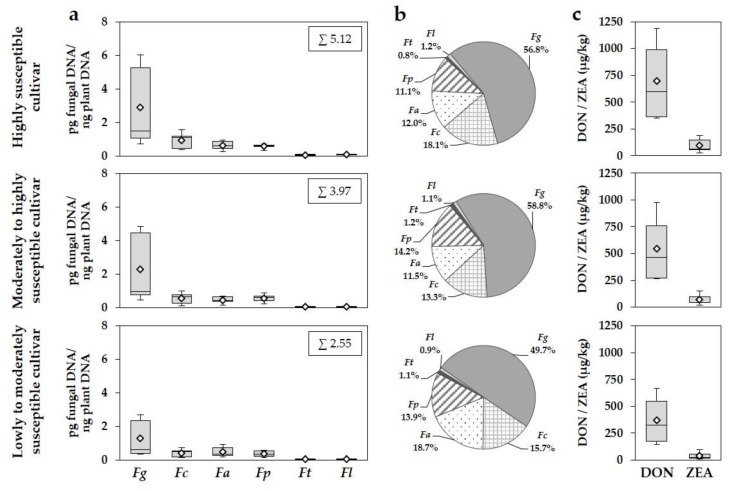
Boxplots and means (white rhombus) of (**a**) DNA amounts of *F. graminearum* (*Fg*), *F. culmorum* (*Fc*), *F. avenaceum* (*Fa*), *F. poae* (*Fp*), *F. tricinctum* (*Ft*) and *F. langsethiae* (*Fl*) (pg fungal DNA/ng plant DNA), (**b**) percentages of DNA amounts of the detected *Fusarium* species to the total *Fusarium* DNA of all detected species and boxplots and means (white rhombus) of (**c**) DON and ZEA concentrations (µg/kg) in wheat grain of the highly, the moderately to highly and the lowly to moderately susceptible cultivar summarized for the years 2013 to 2017 of the seven trial locations (three replications per location) in northern Germany. Five statistics are represented in each boxplot from bottom to top: the smallest observation, lower quartile, median, upper quartile, and largest observation, respectively. *n* = 315.

Averaged over the five monitoring years, the total DNA amount of all detected species was reduced by 22% and 50% in the moderately to highly and the lowly to moderately susceptible cultivar compared to the highly susceptible cultivar (Figure 4a). The averaged DNA amounts of the predominant species *F. graminearum* were reduced by 21% and 56% in the more tolerant cultivars (2.30 and 1.27 pg fungal DNA/ng plant DNA) compared to the highly susceptible cultivar (2.91 pg fungal DNA/ng plant DNA). Especially in years with higher disease pressure (2013, 2017), the cultivation of the lowly to moderately susceptible cultivar significantly reduced the DNA amounts of *F. graminearum* compared to the highly susceptible cultivar, whereas the reduction was less pronounced in the moderately to highly susceptible cultivar (Table 5). Despite the differences in DNA amounts, comparable percentages of DNA amounts of *F. graminearum* to the total *Fusarium* DNA of 56.8% (highly susceptible), 58.8% (moderately to highly susceptible) and 49.7% (lowly to moderately susceptible) were found in the three different susceptible cultivars within the five-year survey (Figure 4b). Following *F. graminearum*, *F. culmorum*, *F. avenaceum* and *F. poae* were the most important species in the FHB complex in all three cultivars summarized for the entire period of study (Figure 4a,b). The highest DNA amounts of *F. culmorum* were found in the highly susceptible cultivar, whereas lower amounts were detected in the more tolerant cultivars (Figure 4a). Significant reductions of DNA amounts towards the highly susceptible cultivar were observed for the lowly to moderately susceptible cultivar in 2013 and 2017 (Table 5). Similar percentages of DNA amounts of *F. culmorum* were observed in all three cultivars (13.3% to 18.7%) (Figure 4b). *F. avenaceum* and also *F. poae* reached comparable DNA amounts between the three different susceptible cultivars averaged for all years (Figure 4a) and within a year (Table 5). The percentages of DNA amounts of both species were similar between cultivars (*F. avenaceum*: 11.5% to 18.7%, *F. poae*: 11.1% to 14.2%). *F. tricinctum* and *F. langsethiae* occurred at a distinct lower level and played only a subordinate role in the total *Fusarium* complex in all three cultivars and years (Figure 4a,b; Table 5). For *F. avenaceum*, *F. poae*, *F. tricinctum* and *F. langsethiae*, no significant differences in species-specific DNA amounts were found between the different susceptible cultivars within a year (Table 5).

DON and ZEA concentrations were also reduced by cultivating more tolerant cultivars (Figure 4c). In the lowly to moderately cultivar, the DON and ZEA concentrations (371 µg DON/kg, 41 µg ZEA/kg) were reduced by 47% and 57%, in the moderately to highly susceptible cultivar by 21% and 26% (548 µg DON/kg, 71 µg ZEA/kg) compared to the highly susceptible cultivar (698 µg DON/kg, 96 µg ZEA/kg). The European maximum levels for DON (1250 µg/kg) and ZEA (100 µg/kg) were exceeded less frequently in the lowly to moderately (DON: 0% of all grain samples; ZEA: 11%) and moderately to highly susceptible cultivar (DON: 10%; ZEA: 23%) compared to the highly susceptible cultivar (DON: 20%; 31%) within the five-year comparison. Significantly lower DON and ZEA concentrations were analysed in years with higher infection pressure for the lowly to moderately susceptible cultivar (Table 5).

### 3.4. Relationship between Meteorological Variables, DNA Amounts, and DON and ZEA Concentrations

**Figure 5:**
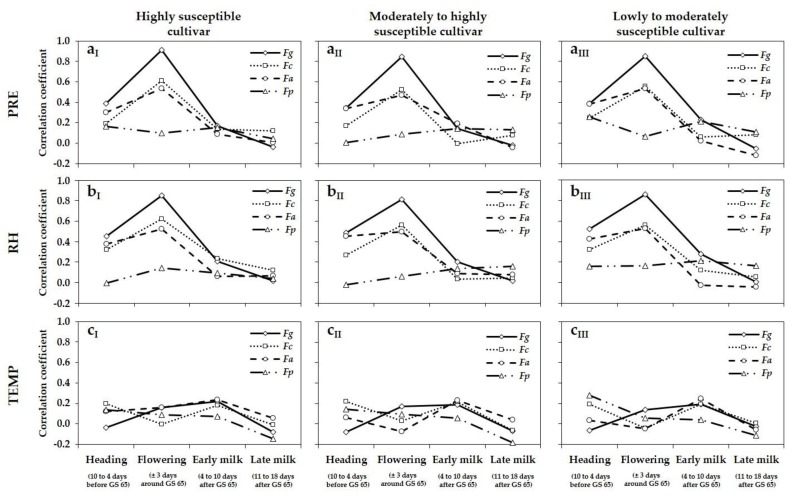
Curves of correlation coefficients (*r*) between meteorological variables (**a**) PRE (cumulative precipitation, mm), (**b**) RH (relative humidity, %) and (**c**) TEMP (mean temperature, °C) 10 to 4 days before GS 65 (heading), ± 3 days around GS 65 (flowering), 4 to 10 days after GS 65 (early milk stage) and 11 to 18 days after GS 65 (late milk stage) and DNA amounts (pg fungal DNA/ng plant DNA) of *F. graminearum* (*Fg*), *F. culmorum* (*Fc*), *F. avenaceum* (*Fa*) and *F. poae* (*Fp*) in wheat grain of the highly (I), the moderately to highly (II) and the lowly to moderately susceptible cultivar (III) and of the seven trial locations (three replications per location) in northern Germany summarized for the years 2013 to 2017. *n* = 315.

**Figure 6:**
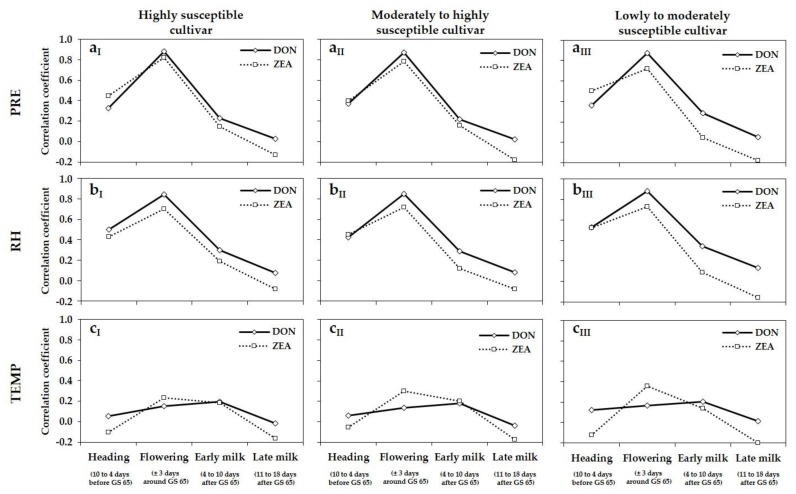
Curves of correlation coefficients (*r*) between meteorological variables (**a**) PRE (cumulative precipitation, mm), (**b**) RH (relative humidity, %) and (**c**) TEMP (mean temperature, °C) 10 to 4 days before GS 65 (heading), ± 3 days around GS 65 (flowering), 4 to 10 days after GS 65 (early milk stage) and 11 to 18 days after GS 65 (late milk stage) and DON and ZEA concentrations (µg/kg) in wheat grain of the highly (I), the moderately to highly (II) and the lowly to moderately susceptible cultivar (III) of the seven trial locations (three replications per location) in northern Germany summarized for the years 2013 to 2017. *n* = 315.

The relationship between meteorological variables (PRE, RH, TEMP) and DNA amounts of the main FHB species *F. graminearum*, *F. culmorum*, *F. avenaceum*, *F. poae*, and DON and ZEA concentrations of the highly, moderately to highly and lowly to moderately susceptible cultivar were established for the periods 10 to 4 days before GS 65 (heading), ± 3 days around GS 65 (flowering), 4 to 10 days after GS 65 (early milk stage) and 11 to 18 days after GS 65 (late milk stage) (Figure 5 and Figure 6). For the highly susceptible cultivar, highly positive correlations were found between the meteorological variables PRE and RH, and DNA amounts of *F. graminearum* (PRE: *r* = 0.91; RH: *r* = 0.85), DON (PRE: *r* = 0.88; RH: *r* = 0.84) and ZEA concentrations (PRE: *r* = 0.82; RH: *r* = 0.76) during the period ± 3 days around GS 65 (flowering) (Figure 5a_I_,b_I_ and Figure 6a_I_,b_I_). The corresponding correlation coefficients for the period 10 to 4 days before GS 65 (heading) were distinctly lower. Especially for the two post-anthesis periods 4 to 10 days after GS 65 (early milk) and 11 to 18 days after GS 65 (late milk), no relationship was found between PRE, RH and DNA amounts of *F. graminearum* and DON and ZEA concentrations. Compared to *F. graminearum*, weaker positive correlations were observed between the meteorological variables PRE and RH and DNA amounts of *F. culmorum* (PRE: *r* = 0.61; RH: *r* = 0.62) and *F. avenaceum* (PRE: *r* = 0.54; RH: *r* = 0.52) ±3 days around the mean flowering date (GS 65). The *r* values were distinctly lower for the pre- and post-flowering periods. No relationships were found between DNA amounts of *F. poae* and PRE and RH for all four periods. For the meteorological variable TEMP and DNA amounts of *F. graminearum*, *F. culmorum*, *F. avenaceum*, *F. poae* as well as DON and ZEA concentrations, no correlations could be established for all considered periods of wheat development (Figure 5c_I_ and Figure 6c_I_). As shown for the highly susceptible cultivar (Figure 5a_I_,b_I_,c_I_ and Figure 6a_I_,b_I_,c_I_), comparable relationships between the meteorological variables PRE, RH and TEMP and DNA amounts of *F. graminearum*, *F. culmorum*, *F. avenaceum*, *F. poae*, as well as DON and ZEA concentrations were observed for the moderately to highly (Figure 5a_II_,b_II_,c_II_ and Figure 6a_II_,b_II_,c_II_) and the lowly to moderately susceptible cultivar (Figure 5a_III_,b_III_,c_III_ and Figure 6a_III_,b_III_,c_III_). For the more tolerant cultivars, the highest positive correlations were also found between the moisture variables PRE and RH, and DNA amounts of *F. graminearum*, DON and ZEA concentrations during the period ±3 days around GS 65 (flowering) with comparable *r* values as shown before for the highly susceptible cultivar. Weaker positive correlations were also established for DNA amounts of *F. culmorum* and *F. avenaceum* and PRE and RH during flowering. For the pre- and post-flowering periods, all relationships between meteorological variables PRE and RH and DNA amounts and mycotoxin concentrations were only weakly or not correlated.

### 3.5. Relationship between DNA Amounts and DON and ZEA Concentrations

The DON and ZEA concentrations of wheat grain of the three different susceptible cultivars within the five-year survey were highly positive correlated to the quantitative detection of the fungal DNA of *F. graminearum* as shown by the correlation coefficients (*r*) in Table 6. Although *F. culmorum* is a known DON and ZEA producer, the overall correlation between DNA amounts and DON and ZEA concentrations was relatively weak compared to *F. graminearum* (Table 6). 

## 4. Discussion

The present study reports the results of a five-year survey from 2013 to 2017 on the composition and quantitative predominance of species belonging to the genus *Fusarium* by quantifying DNA amounts as well as DON and ZEA concentrations in harvested grain samples of winter wheat, depending on cultivar susceptibility and meteorological factors at seven locations in the northernmost federal state of Germany, Schleswig-Holstein. This state is a suitable growing area with large acreages of winter wheat and maritime weather conditions, which are conducive for FHB infections, being a representative region for these analyses. 

In our study, it characterized the *Fusarium* community in 315 grain samples. Using traditional culturing methods for this large number of samples is extremely laborious, requires taxonomical expertise, is time expensive, and increases the risk of overestimating fast-growing species. Culturing methods based on the stimulation of fungal growth from grain on agar plates and subsequent analysis of the different FHB species by morphological determination or qualitative PCR of mycelium outgrowing from the *Fusarium* colonized grain only provide the incidence of the different FHB species by determination of the percentage of grain infected by the different *Fusarium* species [11,30,31]. Furthermore, the visual assessment of damaged grain at harvest or the quantitative detection of mycotoxins only estimate disease symptoms or mycotoxin contamination produced by several *Fusarium* species, but not pathogen populations [30]. Many studies analysing the regional and year-specific occurrence of FHB or the effects of meteorological and agronomic factors focus on parameters such as DON contamination [22,25,26,27,28,35,45,46,47], FHB-damaged grain [28,46,47], incidence and severity of FHB-affected heads and spikelets [25,26,28,45,46,47] or incidence of individual species in wheat grain or heads by culturing methods with the subsequent morphological [12,45,46,48,49] or molecular (qualitative PCR) [50,51,52] determination. The quantitative PCR (qPCR) method used in our study allowed the detection and quantification of species present in very low amounts in wheat grain, in contrast to traditional culturing methods, where the presence of especially slow-growing species will be underestimated. Furthermore, this method reveals the true dominance of the individual species in the FHB complex in wheat grain, which is of paramount importance, due to differences in pathogenicity, environmental requirements, toxin-production abilities, and fungicide sensitivity [10,11,31]. 

Regarding FHB populations found in wheat grain, the situation was very similar to other European regions from a qualitative point of view [1,2,10,11,12,13,31,48,49,51,52,53,54,55,56,57,58,59,60], with the FHB complex mainly composed of *F. graminearum*, *F. culmorum*, *F. avenaceum* and *F. poae*. In addition, *F. tricinctum* and *F. langsethiae* were detected, which were also found in other European surveys. *F. sporotrichioides* and *F. equiseti* were not detected, although a sporadic occurrence could be observed in wheat in some regions of Europe [2,11,12,54,55,56,57]. Within the five-year survey *F. graminearum*, *F. culmorum*, *F. avenaceum* and *F. poae* were found in ≥ 80% of all samples through the entire period of study in the highly susceptible cultivar. High percentages of infected grain samples were also detected for *F. tricinctum* and *F. langsethiae*. In contrast to this qualitative assertion, DNA amounts of the abovementioned species differed considerably between years and especially between species within a year. *F. graminearum* was the most dominant species in all years followed by *F. culmorum*, *F. avenaceum* and *F. poae*, while *F. tricinctum* and *F. langsethiae* played only a very subordinate role in the FHB complex. *F. graminearum* reached the highest percentages of the total *Fusarium* DNA of all detected species, with maximum percentages of up to 70% in years favourable for the development of the FHB disease, averaging nearly 60% for the entire period of study. 

Despite the fact that the wet and cool maritime conditions that are generally encountered in the area surveyed would seem to favour infections by *F. culmorum*, *F. graminearum* was generally the most prevalent species in wheat grain samples in terms of DNA amounts over the entire period of study. Traditionally, *F. culmorum* is associated with cool, wet and humid conditions, while *F. graminearum* dominates in regions with warm and humid environments [18,19]. Not only in Germany, but also in many other countries of Europe, *F. graminearum* becomes the major FHB constituent expanding towards regions with cooler and maritime conditions, replacing *F. culmorum* as the main causal agent of FHB in the past [10,11,49,53,54,56,58,59]. The emergence of *F. graminearum* has been often linked to an increased production of maize, another primary host of *F. graminearum* in contrast to *F. culmorum* [10,11,13,48]. Maize crop residues promote the mass production of ascospores of *F. graminearum*, which appear to greatly outnumber conidia and which can travel over long distances [14,15,16,17,61]. In contrast, *F. culmorum* only produce asexual conidia, which are mainly dispersed by rain splashing [2,14]. Additionally, in the area surveyed a dramatic increase in maize (for bioenergy) was observed by 147%, from 79,200 ha in 2000 [62] to 195,600 ha in 2011, with an unchanged cultivation area of wheat with 210,300 ha (arable crop area in 2011: 673,300 ha) [63]. Although wheat grain samples within our survey originated from maize-free crop rotations, *F. graminearum* was the predominant species in all years, indicating that the increased production area of maize is likely to be responsible for an increased infection potential of wheat with *F. graminearum* in this area. Furthermore, the shift toward more *F. graminearum* may involve an adaption to cooler climate, changes in climate toward warmer and more humid springs/summers and a superior competitiveness over other *Fusarium* species [10]. Although *F. graminearum* dominated over *F. culmorum*, a positive correlation of DNA amounts was found between these two species, indicating that they were favoured by similar meteorological conditions. This is in contrast to other reports [49,51], which found no or a negative interaction between *F. graminearum* and *F. culmorum*.

In addition to *F. graminearum* and *F. culmorum*, *F. avenaceum* and *F.* poae are often represented in the *Fusarium* complex as causative agents of FHB in Europe, reaching partially high frequencies of infected grain or DNA amounts in wheat grain [2,10,11,12,31,48,49,52,54,56,57,58]. Like *F. culmorum*, *F. avenaceum* is associated with cool, wet and humid conditions, and is therefore often found as one of the predominant species in the FHB complex, mainly in northern Europe [11,19,31,57,58]. *F. poae* has been described to be associated with warmer and drier conditions with an increasing occurrence in some years and regions of Europe [19,51,54,56]. Both species were not found in greater extent in our five-year survey and DNA amounts were apparently stable over the entire period of study and significantly lower than those of *F. graminearum*. Fernandez and Chen [64] and Xu et al. [10] suggest that *F. graminearum* is probably the most pathogenic FHB species, which rapidly infects wheat heads and developing grain under favourable meteorological conditions, which may preclude the infection by other FHB species. Xu et al. [65,66] described *F. poae* and *F. avenaceum* as less pathogenic than the more aggressive species *F. culmorum* and especially *F. graminearum*. They found that in mixed inoculations, *F. graminearum* was most competitive, whereas *F. poae* was the least competitive of the four species including *F. avenaceum* and *F. culmorum*, which was more competitive than *F. avenaceum*. These findings suggest that *F. avenaceum* and *F. poae* can colonise wheat grain under meteorological conditions less favourable to *F. graminearum*. The dominant and increased occurrence of *F. graminearum* towards *F. avenaceum* and *F. poae* in the area surveyed was particularly observed in 2013 and 2017 under meteorological conditions favourable for *F. graminearum*, while the remaining years 2014 to 2016 were, in general, not conductive for FHB infections. 

Our results show a differential susceptibility response of the three investigated cultivars to FHB infections and DON and ZEA concentrations. Most studies analysing the susceptibility against FHB focus on parameters such as DON content, FHB damaged grain, disease severity of affected heads and spikelets and frequency of individual *Fusarium* species [27,28,49,50,51,67,68], and were often conducted using artificial infections [28,67]. Beyer et al. [27] noted that comparisons of different cultivars grown at different locations, in different years and after different pre-crops would bias a fair assessment of cultivar susceptibility. In our study, cultivar susceptibility was evaluated by measuring both DNA amounts of the co-occurring FHB species as well as DON and ZEA concentrations in wheat grain using natural field infections. Each of the three differently susceptible cultivars (highly, moderately to highly and lowly to moderately susceptible) was grown at each of the seven locations and in all years, which allowed a fair assessment between cultivars. Furthermore, soil cultivation and previous crop were the same at a location in each year and should have had no effect on FHB occurrence and mycotoxin contamination, which is in line with other authors [14,27,50]. The FHB favourable reduced cultivation tillage was only applied after oilseed rape (non-host for FHB), ploughing was done after pre-crop wheat, burying the wheat residues for interrupting the saprophytic survival of the fungi on wheat stubbles. The ranking of the three cultivars in the five-year survey was similar between years and within a year, with the highly susceptible cultivar as the most susceptible and the lowly to moderately susceptible cultivar as the most tolerant cultivar. The same FHB species complex was found in all cultivars with a comparable year-specific quantitative occurrence of the six detected *Fusarium* species as well as DON and ZEA concentrations, but with decreased DNA amounts and mycotoxin contents in the more tolerant cultivars. This was particularly pronounced in years with an increased disease pressure (2013, 2017). In all cultivars, *F. graminearum* was the predominant species, followed by *F. culmorum*, *F. avenaceum* and *F. poae*. The reduction in DNA amounts and mycotoxin concentrations between the three cultivars were mainly caused by the reduced DNA amounts of *F. culmorum* and especially *F. graminearum* in the more tolerant cultivars. Astonishingly, comparable percentages of DNA amounts of the six species to the total *Fusarium* DNA amount of all detected species were found between the three cultivars for each species in all years. Increased cultivar resistance to FHB seems to limit the occurrence of almost all species of the FHB complex. Furthermore, no prevalence of a certain species to any of the tested cultivars was found. Mesterházy et al. [28] also concluded that the degrees of FHB severity of wheat cultivars to different *Fusarium* species, including *F. graminearum*, *F. culmorum*, *F. avenaceum* and *F. poae*, were very similar, indicating that the cultivar resistance to any *Fusarium* species implies a similar level of resistance to other *Fusarium* species. 

Field surveys conducted elsewhere have indicated that the prevalence and severity of FHB varied strongly from year to year and also from location to location [12,48,49,52]. In our five-year survey, DNA amounts as well as DON and ZEA concentrations also varied strongly from year to year and from location to location within a year. One of the major environmental factors influencing FHB occurrence are meteorological conditions, which vary often between growing seasons and locations within a season. Significant precipitation, high relative humidity and warm temperatures, coinciding with flowering of wheat, favour infection and development of FHB [1,18,19]. Within our five-year survey, differences in DNA amounts, especially of *F. graminearum*, and also DON and ZEA concentrations between growing seasons, were affected by the varying meteorological variables PRE and RH during flowering, shown by the highly positive correlations between these meteorological variables and DNA amounts of *F. graminearum* and DON and ZEA concentrations. The highest DNA amounts of the predominant species *F. graminearum* as well as mycotoxin concentrations were detected in 2013 and 2017 in the three different susceptible cultivars. These years were characterized by the highest values of PRE and RH at flowering. In 2014, 2015 and 2016, lower DNA amounts and mycotoxin concentrations were analysed, attributable to the lower values of PRE and RH during this period. The fluctuations of PRE and RH between locations within a year resulted in a large variation of DNA amounts and DON and ZEA concentrations within a year. Positive correlations were also observed between the meteorological variables PRE and RH, and DNA amounts of *F. culmorum* and *F. avenaceum*. Lacey et al. [45] also showed that after artificial field inoculation with conidia of *F. culmorum* at different growth stages of wheat, most head infection and DON contamination was restricted to the period of flowering, whereas little or no symptoms as well as lower DON concentrations resulted from inoculations before (head emergence) and after flowering (early and late milk stage). Similar results were obtained by Rossi et al. [30], who inoculated wheat heads with conidia of *F. graminearum* and *F. culmorum* under field conditions at six growth stages between heading and dough ripening. Inoculations at flowering resulted in the highest incidences of infected grain and DON concentrations compared to inoculations before and after flowering. In contrast, Del Ponte et al. [46] and Yoshida and Nakajima [47] showed in their studies with artificial inoculations at different growth stages in the greenhouse that heavy FHB infections associated with high mycotoxin concentrations are still possible during the milk stage and often resulted in higher values of FHB damaged grain and mycotoxin concentrations compared to inoculations during flowering. However, our results indicate that under natural field infections, the periods several days before (heading) and after flowering (early and late milk stage) were less important towards FHB infections than during flowering. For the pre- and post-flowering periods the relationships between the meteorological variables PRE and RH and DNA amounts of *F. graminearum*, *F. culmorum* and *F. avenaceum* and mycotoxin concentrations were less correlated than for the period of wheat flowering. There was no evidence of correlations between TEMP and DNA amounts of any *Fusarium* species and DON and ZEA concentrations during flowering. For wheat, wetness periods of at least 24 h and temperatures above 15 °C are required for significant infection by *F. graminearum*, *F. culmorum*, *F. avenaceum* and *F. poae* [18]. In almost all years, TEMP during flowering was higher than this critical value and does not seem to be a limiting factor, although higher optimal temperature requirements were described for several *Fusarium* species [18,69], which will never be reached under field conditions in the area surveyed. 

The often co-occurring mycotoxins DON and ZEA are the most common mycotoxins in wheat, associated with *F. graminearum* and *F. culmorum* [2,4,8]. In our five-year survey, high levels of DON and ZEA were detected in wheat grain samples in years with a strong occurrence of these two species, especially with *F. graminearum*. Correlation results between DNA amounts and DON and ZEA concentrations indicate that *F. graminearum* is the main producer of DON and ZEA in wheat grain samples. Grain samples were not analysed for DON derivates 3-AcDON, 15-AcDON and NIV, which can be produced by the two aforementioned species [2,4]. In addition to *F. graminearum* and *F. culmorum*, other toxigenic species present in grain samples are capable to produce several mycotoxins. *F. avenaceum* is known to produce MON, BEA and ENS, *F. poae* to produce NIV and BEA. *F. langsethiae* has been described as a producer of the highly toxic HT-2 and T-2 toxins, which can also be synthesized by a few isolates of *F. poae*. *F. tricinctum* is able to produce MON and ENS [2,4,8,54,70]. We did not measure the concentrations of MON, BEA, ENS, NIV, HT-2 and T-2 toxin, but the range of *Fusarium* species present in grain samples indicates a potential for the contamination with these mycotoxins as shown in others studies [54,56]. It must be noticed that *F. avenaceum* and *F. poae* often, and *F. tricinctum* and *F. langsethiae* in general occurred with very small DNA amounts in wheat grain, indicating that these species only produce small or very small amounts of the abovementioned mycotoxins. Due to the strong occurrence of *F. graminearum* in wheat growing areas, a contamination with DON and ZEA in wheat grain can be expected.

## Figures and Tables

**Table 1:** Coordinates and agronomic practices (crop rotation, previous crop, soil cultivation) of the seven trial locations of the regional *Fusarium* monitoring in winter wheat in northern Germany from 2013 to 2017. Coordinate System: ETRS89 (European Terrestrial Reference System 1989). OR = Oilseed rape, SB = Sugar beet, WW = Winter wheat, WB = Winter barley.

Trial Location	Coordinates (ETRS89)		Crop Rotation	Previous Crop	Soil Cultivation
	xETRS	yETRS			
Barlt	1004362	7173698	SB-WW-WW	WW	Plough
Elskop	1060012	7133704	OR-WW-WW	WW	Plough
Futterkamp	1183896	7225601	OR-WW-WB	OR	Reduced tillage
Kastorf	1175513	7123486	OR-WW-WW	WW	Plough
Kluvensiek	1092115	7234219	OR-WW-WB	OR	Reduced tillage
Loit	1080326	7286046	OR-WW-WB	OR	Reduced tillage
Sönke-Nissen-Koog	987384	7291393	OR-WW-WW	WW	Plough

**Table 2:** Percentages of wheat grain samples infected with *F. graminearum* (*Fg*), *F. culmorum* (*Fc*), *F. avenaceum* (*Fa*), *F. poae* (*Fp*), *F. tricinctum* (*Ft*) and *F. langsethiae* (*Fl*) of the highly susceptible cultivar of the seven trial locations (three replications per location) in northern Germany from 2013 to 2017 and across years. *n* = 105.

Species	2013	2014	2015	2016	2017	2013–2017
***Fg***	100	76	57	71	100	81
***Fc***	100	71	57	100	100	86
***Fa***	100	67	71	62	100	80
***Fp***	100	81	52	95	100	86
***Ft***	71	67	48	67	81	67
***Fl***	76	62	52	57	76	65

**Table 3:** ANOVA results for the dependent variables DNA amount (pg fungal DNA/ng plant DNA), DON and ZEA concentration (µg/kg) in wheat grain depending on year (Y), cultivar (Cv), species (Spp; only DNA amount) and their interactions summarized for all survey years (2013–2017), cultivars (highly, moderately to highly and lowly to moderately susceptible) and detected *Fusarium* species (*F. graminearum*, *F. culmorum*, *F. avenaceum*, *F. poae*, *F. tricinctum*, *F. langsethiae*) of the seven trial locations (three replications per location) in northern Germany. *n* = 315.

Effect	*df*	DNA Amount (pg Fungal DNA/ng Plant DNA)		DON (µg/kg)		ZEA (µg/kg)	
		*F*	*p*	*F*	*p*	*F*	*p*
**Year (Y)**	4	5.315	**0.0003**	154.317	**<0.0001**	129.643	**<0.0001**
**Cultivar (Cv)**	2	8.932	**0.0042**	48.486	**<0.0001**	64.051	**<0.0001**
**Species (Spp)**	5	128.769	**<0.0001**				
**Y × Cv**	8	1.328	0.2250	3.134	**0.0022**	5.740	**<0.0001**
**Y × Spp**	20	19.284	**<0.0001**				
**Cv × Spp**	10	8.453	**<0.0001**				
**Y × Cv × Spp**	40	2.317	**<0.0001**				

Note: The numbers of probability (*p*) in the case of significant effects (*p* ≤ 0.05) are written in bold letters.

**Table 4:** Correlation coefficients (*r*) of DNA amounts in wheat grain between *F. graminearum* (*Fg*), *F. culmorum* (*Fc*), *F. avenaceum* (*Fa*), *F. poae* (*Fp*), *F. tricinctum* (*Ft*) and *F. langsethiae* (*Fl*) summarized for all survey years (2013–2017) and cultivars (highly, moderately to highly and lowly to moderately susceptible) of the seven trial locations (three replications per location) in northern Germany. *n* = 315.

	*Fa*	*Fc*	*Fg*	*Fl*	*Fp*
***Fc***	0.562				
***Fg***	0.294	0.485			
***Fl***	0.285	0.044	0.065		
***Fp***	0.198	0.180	0.123	0.246	
***Ft***	0.287	0.079	0.056	0.284	0.473

**Table 5:** Mean DNA amounts (± *SD*) of *F. graminearum* (*Fg*), *F. culmorum* (*Fc*), *F. avenaceum* (*Fa*), *F. poae* (*Fp*), *F. tricinctum* (*Ft*) and *F. langsethiae* (*Fl*) (pg fungal DNA/ng plant DNA) and mean concentrations (± *SD*) of DON and ZEA (µg/kg) in wheat grain of the highly, the moderately to highly and the lowly to moderately susceptible cultivar of the seven trial locations (three replications per location) in northern Germany from 2013 to 2017. Mean values labelled with the same letter are not significantly different from each other (*p* > 0.05). Different letters describe differences between the three cultivars for each *Fusarium* species, DON and ZEA concentrations within a year. *n* = 315.

	Cultivar Susceptibility	DNA Amount (pg Fungal DNA/ng Plant DNA)						Mycotoxin Content (µg/kg)	
		*Fg*	*Fc*	*Fa*	*Fp*	*Ft*	*Fl*	DON	ZEA
**2017**	**High**	5.25 ± 3.70 a	1.08 ± 0.55 a	0.88 ± 0.34 a	0.54 ± 0.43 a	0.05 ± 0.03 a	0.08 ± 0.08 a	987 ± 438 a	146 ± 66 a
	**Moderate to high**	4.46 ± 3.53 ab	0.64 ± 0.40 ab	0.68 ± 0.40 a	0.42 ± 0.33 a	0.07 ± 0.03 a	0.04 ± 0.03 a	761 ± 357 a	99 ± 50 a
	**Low to moderate**	2.34 ± 1.41 b	0.48 ± 0.29 b	0.72 ± 0.46 a	0.33 ± 0.25 a	0.03 ± 0.04 a	0.02 ± 0.03 a	546 ± 226 b	54 ± 26 b
**2016**	**High**	1.48 ± 1.93 a	1.16 ± 1.32 a	0.58 ± 0.94 a	0.73 ± 0.55 a	0.07 ± 0.09 a	0.03 ± 0.04 a	607 ± 392 a	54 ± 49 a
	**Moderate to high**	0.96 ± 1.44 a	0.79 ± 0.86 a	0.38 ± 0.37 a	0.89 ± 0.64 a	0.09 ± 0.11 a	0.02 ± 0.03 a	463 ± 324 ab	40 ± 42 a
	**Low to moderate**	0.61 ± 0.99 a	0.47 ± 0.89 a	0.32 ± 0.59 a	0.53 ± 0.53 a	0.02 ± 0.03 a	0.01 ± 0.02 a	308 ± 252 b	22 ± 25 a
**2015**	**High**	0.69 ± 1.40 a	0.38 ± 0.42 a	0.44 ± 0.52 a	0.33 ± 0.56 a	0.04 ± 0.05 a	0.05 ± 0.05 a	356 ± 103 a	29 ± 22 a
	**Moderate to high**	0.46 ± 0.56 a	0.18 ± 0.16 a	0.42 ± 0.53 a	0.22 ± 0.30 a	0.04 ± 0.06 a	0.05 ± 0.05 a	273 ± 71 a	18 ± 13 a
	**Low to moderate**	0.34 ± 0.61 a	0.17 ± 0.29 a	0.27 ± 0.37 a	0.14 ± 0.22 a	0.02 ± 0.04 a	0.04 ± 0.05 a	113 ± 75 a	11 ± 8 a
**2014**	**High**	1.08 ± 1.27 a	0.45 ± 0.87 a	0.26 ± 0.28 a	0.60 ± 0.64 a	0.01 ± 0.01 a	0.07 ± 0.02 a	366 ± 283 a	62 ± 42 a
	**Moderate to high**	0.75 ± 1.03 a	0.29 ± 0.42 a	0.14 ± 0.16 a	0.61 ± 0.86 a	0.02 ± 0.02 a	0.03 ± 0.02 a	267 ± 193 a	44 ± 30 a
	**Low to moderate**	0.37 ± 0.71 a	0.15 ± 0.27 a	0.16 ± 0.10 a	0.23 ± 0.28 a	0.01 ± 0.01 a	0.01 ± 0.02 a	131 ± 98 a	27 ± 17 a
**2013**	**High**	6.04 ± 2.69 a	1.57 ± 0.60 a	0.93 ± 0.59 a	0.64 ± 0.44 a	0.05 ± 0.06 a	0.09 ± 0.06 a	1186 ± 344 a	189 ± 91 a
	**Moderate to high**	4.85 ± 2.13 ab	1.01 ± 0.63 ab	0.66 ± 0.39 a	0.68 ± 0.39 a	0.03 ± 0.03 a	0.07 ± 0.04 a	975 ± 212 a	154 ± 58 a
	**Low to moderate**	2.68 ± 1.18 b	0.73 ± 0.58 b	0.90 ± 0.87 a	0.54 ± 0.56 a	0.06 ± 0.12 a	0.03 ± 0.03 a	669 ± 116 b	95 ± 34 b

**Table 6:** Correlation coefficients (*r*) between DNA amounts (pg fungal DNA/ng plant DNA) of *F. graminearum* (*Fg*) and *F. culmorum* (*Fc*) and DON and ZEA concentrations (µg/kg) in wheat grain of the highly, the moderately to highly and the lowly to moderately susceptible cultivar summarized for the years 2013 to 2017. *n* = 315.

Species	Mycotoxin	Cultivar Susceptibility		
		High	Moderate to High	Low to Moderate
***Fg***	**DON**	0.911	0.883	0.889
***Fc***	**DON**	0.645	0.622	0.589
***Fg***	**ZEA**	0.877	0.832	0.854
***Fc***	**ZEA**	0.497	0.596	0.418

## Supplementary Materials

The following are available online at https://www.mdpi.com/2076-2607/8/4/617/s1, Table S1: Percentages of wheat grain samples infected with *F. graminearum* (*Fg*), *F. culmorum* (*Fc*), *F. avenaceum* (*Fa*), *F. poae* (*Fp*), *F. tricinctum* (*Ft*) and *F. langsethiae* (*Fl*) of the moderately to highly susceptible cultivar of the seven trial locations (three replications per location) in northern Germany from 2013 to 2017 and across years. *n* = 105; Table S2: Percentages of wheat grain samples infected with *F. graminearum* (*Fg*), *F. culmorum* (*Fc*), *F. avenaceum* (*Fa*), *F. poae* (*Fp*), *F. tricinctum* (*Ft*) and *F. langsethiae* (*Fl*) of the lowly to moderately susceptible cultivar of the seven trial locations (three replications per location) in northern Germany from 2013 to 2017 and across years. *n* = 105; Figure S1: Boxplots and means (white rhombus) of (a) DNA amounts of *F. graminearum* (*Fg*), *F. culmorum* (*Fc*), *F. avenaceum* (*Fa*), *F. poae* (*Fp*), *F. tricinctum* (*Ft*) and *F. langsethiae* (*Fl*) (pg fungal DNA/ng plant DNA), (b) percentages of DNA amounts of the detected *Fusarium* species to the total *Fusarium* DNA amount of all detected species and boxplots and means (white rhombus) of (c) DON and ZEA concentrations (µg/kg) in wheat grain of the moderately to highly susceptible cultivar of the seven trial locations (three replications per location) in northern Germany from 2013 to 2017. Five statistics are represented in each boxplot from bottom to top: the smallest observation, lower quartile, median, upper quartile, and largest observation, respectively. Mean values labelled with the same letter (small or big) are not significantly different from each other (*p* > 0.05). Capital letters describe differences for one species and DON and ZEA concentrations between years and small letters differences between the detected Fusarium species within a year. *n* = 105; Figure S2: Boxplots and means (white rhombus) of (a) DNA amounts of *F. graminearum* (*Fg*), *F. culmorum* (*Fc*), *F. avenaceum* (*Fa*), *F. poae* (*Fp*), *F. tricinctum* (*Ft*) and *F. langsethiae* (*Fl*) (pg fungal DNA/ng plant DNA), (b) percentages of DNA amounts of the detected *Fusarium* species to the total *Fusarium* DNA amount of all detected species and boxplots and means (white rhombus) of (c) DON and ZEA concentrations (µg/kg) in wheat grain of the moderately to highly susceptible cultivar of the seven trial locations (three replications per location) in northern Germany from 2013 to 2017. Five statistics are represented in each boxplot from bottom to top: the smallest observation, lower quartile, median, upper quartile, and largest observation, respectively. Mean values labelled with the same letter (small or big) are not significantly different from each other (*p* > 0.05). Capital letters describe differences for one species and DON and ZEA concentrations between years and small letters differences between the detected Fusarium species within a year. *n* = 105.

## References

[1] Parry D.W., Jenkinson P., McLeod L. (1995). *Fusarium* ear blight (scab) in small grain cereals-a review. Plant Pathol..

[2] Bottalico A., Perrone G. (2002). Toxigenic *Fusarium* species and mycotoxins associated with head blight in small-grain cereals in Europe. Eur. J. Plant Pathol..

[3] Logrieco A., Mulè G., Moretti A., Bottalico A. (2002). Toxigenic *Fusarium* species and mycotoxins associated with maize ear rot in Europe. Eur. J. Plant Pathol..

[4] Ferrigo D., Raiola A., Causin R. (2016). *Fusarium* toxins in cereals: Occurrence, legislation, factors promoting the appearance and their management. Molecules.

[5] Edwards S.G. (2004). Influence of agricultural practices on fusarium infection of cereals and subsequent contamination of grain by trichothecene mycotoxins. Toxicol. Lett..

[6] Osborne L.E., Stein J.M. (2007). Epidemiology of *Fusarium* head blight on small-grain cereals. Int. J. Food Microbiol..

[7] Martínez M., Ramírez Albuquerque L., Arata A.F., Biganzoli F., Fernández Pinto V., Stenglein S.A. (2020). Effects of *Fusarium graminearum* and *Fusarium poae* on disease parameters, grain quality and mycotoxins contamination in bread wheat (Part I). J. Sci. Food Agric..

[8] Desjardins A.E. (2006). Fusarium Mycotoxins: Chemistry, Genetics and Biology.

[9] European Commission (2007). Commission Regulation (EC) No 1126/2007of 28 September 2007 amending Regulation (EC) No 1881/2006 setting maximum levels for certain contaminants infoodstuffs as regards Fusarium toxins in maize and maize products. Off. J. Eur. Union.

[10] Xu X.-M., Parry D.W., Nicholson P., Thomsett M.A., Simpson D., Edwards S.G., Cooke B.M., Doohan F.M., Brennan J.M., Moretti A. (2005). Predominance and association of pathogenic fungi causing *Fusarium* ear blight in wheat in four European countries. Eur. J. Plant Pathol..

[11] Nielsen L.K., Jensen J.D., Nielsen G.C., Jensen J.E., Spliid N.H., Thomsen I.K., Justesen A.F., Collinge D.B., Jørgensen L.N. (2011). *Fusarium* head blight of cereals in Denmark: Species complex and related mycotoxins. Phytopathology.

[12] Czaban J., Wróblewska B., Sułek A., Mikos M., Boguszewska E., Podolska G., Nieróbca A. (2015). Colonisation of winter wheat grain by *Fusarium* spp. and mycotoxin content as dependent on a wheat variety, crop rotation, a crop management system and weather conditions. Food Addit. Contam. Part A.

[13] Waalwijk C., Kastelein P., Vries I.d., Kerényi Z., van der Lee T., Hesselink T., Köhl J., Kema G. (2003). Major changes in *Fusarium* spp. in wheat in the Netherlands. Eur. J. Plant Pathol..

[14] Champeil A., Doré T., Fourbet J.F. (2004). *Fusarium* head blight: Epidemiological origin of the effects of cultural practices on head blight attacks and the production of mycotoxins by *Fusarium* in wheat grains. Plant Sci..

[15] Jenkinson P., Parry D.W. (1994). Splash dispersal of conidia of *Fusarium culmorum* and *Fusarium avenaceum*. Mycol. Res..

[16] Maldonado-Ramirez S.L., Schmale D.G., Shields E.J., Bergstrom G.C. (2005). The relative abundance of viable spores of *Gibberella zeae* in the planetary boundary layer suggests the role of long-distance transport in regional epidemics of Fusarium head blight. Agric. For. Meteorol..

[17] Keller M.D., Bergstrom G.C., Shields E.J. (2014). The aerobiology of *Fusarium graminearum*. Aerobiologia.

[18] Doohan F.M., Brennan J., Cooke B.M. (2003). Influence of climatic factors on *Fusarium* species pathogenic to cereals. Eur. J. Plant Pathol..

[19] Xu X.-M., Nicholson P., Thomsett M.A., Simpson D., Cooke B.M., Doohan F.M., Brennan J., Monaghan S., Moretti A., Mule G. (2008). Relationship between the fungal complex causing *Fusarium* head blight of wheat and environmental conditions. Phytopathology.

[20] Bai G., Shaner G. (2004). Management and resistance in wheat and barley to *Fusarium* head blight. Annu. Rev. Phytopathol..

[21] Hooker D.C., Schaafsma A.W., Tamburic-Ilincic L. (2002). Using weather variables pre- and post-heading to predict deoxynivalenol content in winter wheat. Plant Dis..

[22] Franz E., Booij K., van der Fels-Klerx I. (2009). Prediction of deoxynivalenol content in dutch winter wheat. J. Food Prot..

[23] Váňová M., Klem K., Matušinský P., Trnka M. (2010). Prediction model for deoxynivalenol in wheat grain based on weather conditions. Plant Protect. Sci..

[24] Kriss A.B., Paul P.A., Xu X., Nicholson P., Doohan F.M., Hornok L., Rietini A., Edwards S.G., Madden L.V. (2012). Quantification of the relationship between the environment and *Fusarium* head blight, *Fusarium* pathogen density, and mycotoxins in winter wheat in Europe. Eur. J. Plant Pathol..

[25] Dill-Macky R., Jones R.K. (2000). The effect of previous crop residues and tillage on *Fusarium* head blight of wheat. Plant Dis..

[26] Schaafsma A.W., Tamburic-Ilincic L., Hooker D.C. (2005). Effect of previous crop, tillage, field size, adjacent crop, and sampling direction on airborne propagules of *Gibberella zeae*/*Fusarium graminearum*, fusarium head blight severity, and deoxynivalenol accumulation in winter wheat. Can. J. Plant Pathol..

[27] Beyer M., Klix M.B., Klink H., Verreet J.-A. (2006). Quantifying the effects of previous crop, tillage, cultivar and triazole fungicides on the deoxynivalenol content of wheat grain—A review. J. Plant Dis. Prot..

[28] Mesterházy Á., Bartók T., Kászonyi G., Varga M., Tóth B., Varga J. (2005). Common resistance to different *Fusarium* spp. causing *Fusarium* head blight in wheat. Eur. J. Plant Pathol..

[29] Zhu Z., Hao Y., Mergoum M., Bai G., Humphreys G., Cloutier S., Xia X., He Z. (2019). Breeding wheat for resistance to *Fusarium* head blight in the Global North: China, USA, and Canada. Crop J..

[30] Rossi V., Terzi V., Moggi F., Morcia C., Faccioli P., Haidukowski M., Pascale M. (2007). Assessment of *Fusarium* infection in wheat heads using a quantitative polymerase chain reaction (qPCR) assay. Food Addit. Contam..

[31] Nicolaisen M., Suproniene S., Nielsen L.K., Lazzaro I., Spliid N.H., Justesen A.F. (2009). Real-time PCR for quantification of eleven individual *Fusarium* species in cereals. J. Microbiol. Methods.

[32] Deutscher Wetterdienst (DWD) Klimareport Schleswig-Holstein. https://www.dwd.de/DE/leistungen/klimareport_sh/download_report_2017.pdf?__blob=publicationFile&v=5.

[33] Statistisches Amt für Hamburg und Schleswig-Holstein Die Bodennutzung in Schleswig-Holstein 2017. http://epub.sub.uni-hamburg.de/epub/volltexte/2018/78253/pdf/C_I_1_j_17_SH_e.pdf.

[34] Verreet J.A., Klink H., Hoffmann G.M. (2000). Regional monitoring for disease prediction and optimization of plant protection measuares: The IPM wheat model. Plant Dis..

[35] Birr T., Verreet J.-A., Klink H. (2019). Prediction of deoxynivalenol and zearalenone in winter wheat grain in a maize-free crop rotation based on cultivar susceptibility and meteorological factors. J. Plant Dis. Prot..

[36] Bundessortenamt Beschreibende Sortenliste. https://www.bundessortenamt.de/bsa/media/Files/BSL/bsl_getreide_2008.pdf.

[37] Zadoks J.C., Chang T.T., Konzak C.F. (1974). A decimal code for the growth stages of cereals. Weed Res..

[38] Hothorn T., Bretz F., Westfall P., Heiberger R.M., Schuetzenmeister A., Scheibe S. Multcomp: Simultaneous Inference in General Parametric Models. R Package Version 1.4-10. https://cran.r-project.org/src/contrib/Archive/multcomp/.

[39] Pinheiro J., Bates D., DebRoy S., Sarkar D. Nlme: Linear and Nonlinear Mixed Effects Models. R Package Version 3.1-140. https://cran.r-project.org/src/contrib/Archive/nlme/.

[40] Laird N.M., Ware J.H. (1982). Random-effects models for longitudinal data. Biometrics.

[41] Verbeke G., Molenberghs G. (2000). Linear Mixed Models for Longitudinal Data.

[42] Nakagawa S., Schielzeth H. (2013). A general and simple method for obtaining *R^2^* from generalized linear mixed-effects models. Methods Ecol. Evol..

[43] Schaarschmidt F., Vaas L. (2009). Analysis of trials with complex treatment structure using multiple contrast tests. HortScience.

[44] Bretz F., Hothorn T., Westfall P.H. (2011). Multiple Comparisons Using R.

[45] Lacey J., Bateman G.L., Mirocha C.J. (1999). Effects of infection time and moisture on development of ear blight and deoxynivalenol production by *Fusarium* spp. in wheat. Ann. Appl. Biol..

[46] Del Ponte E.M., Fernandes J.M.C., Bergstrom G.C. (2007). Influence of growth stage on *Fusarium* head blight and deoxynivalenol production in wheat. J. Phytopathol..

[47] Yoshida M., Nakajima T. (2010). Deoxynivalenol and nivalenol accumulation in wheat infected with *Fusarium graminearum* during grain development. Phytopathology.

[48] Isebaert S., de Saeger S., Devreese R., Verhoeven R., Maene P., Heremans B., Haesaert G. (2009). Mycotoxin-producing *Fusarium* species occurring in winter wheat in Belgium (Flanders) during 2002–2005. J. Phytopathol..

[49] Chandelier A., Nimal C., André F., Planchon V., Oger R. (2011). *Fusarium* species and DON contamination associated with head blight in winter wheat over a 7-year period (2003–2009) in Belgium. Eur. J. Plant Pathol..

[50] Klix M.B., Beyer M., Verreet J.-A. (2008). Effects of cultivar, agronomic practices, geographic location, and meteorological conditions on the composition of selected *Fusarium* species on wheat heads. Can. J. Plant Pathol..

[51] Audenaert K., van Broeck R., Bekaert B., de Witte F., Heremans B., Messens K., Höfte M., Haesaert G. (2009). *Fusarium* head blight (FHB) in Flanders: Population diversity, inter-species associations and DON contamination in commercial winter wheat varieties. Eur. J. Plant Pathol..

[52] Giraud F., Pasquali M., El Jarroudi M., Vrancken C., Brochot C., Cocco E., Hoffmann L., Delfosse P., Bohn T. (2010). *Fusarium* head blight and associated mycotoxin occurrence on winter wheat in Luxembourg in 2007/2008. Food Addit. Contam. Part A.

[53] Talas F., Parzies H.K., Miedaner T. (2011). Diversity in genetic structure and chemotype composition of *Fusarium graminearum* sensu stricto populations causing wheat head blight in individual fields in Germany. Eur. J. Plant Pathol..

[54] Lindblad M., Gidlund A., Sulyok M., Börjesson T., Krska R., Olsen M., Fredlund E. (2013). Deoxynivalenol and other selected *Fusarium* toxins in Swedish wheat - Occurrence and correlation to specific *Fusarium* species. Int. J. Food Microbiol..

[55] Covarelli L., Beccari G., Prodi A., Generotti S., Etruschi F., Juan C., Ferrer E., Mañes J. (2015). *Fusarium* species, chemotype characterisation and trichothecene contamination of durum and soft wheat in an area of central Italy. J. Sci. Food Agric..

[56] Hellin P., Dedeurwaerder G., Duvivier M., Scauflaire J., Huybrechts B., Callebaut A., Munaut F., Legrève A. (2016). Relationship between *Fusarium* spp. diversity and mycotoxin contents of mature grains in southern Belgium. Food Addit. Contam. Part A.

[57] Hietaniemi V., Rämö S., Yli-Mattila T., Jestoi M., Peltonen S., Kartio M., Sieviläinen E., Koivisto T., Parikka P. (2016). Updated survey of *Fusarium* species and toxins in Finnish cereal grains. Food Addit. Contam. Part A.

[58] Hofgaard I.S., Aamot H.U., Torp T., Jestoi M., Lattanzio V.M.T., Klemsdal S.S., Waalwijk C., van der Lee T., Brodal G. (2016). Associations between *Fusarium* species and mycotoxins in oats and spring wheat from farmers’ fields in Norway over a six-year period. World Mycotoxin J..

[59] Karlsson I., Friberg H., Kolseth A.-K., Steinberg C., Persson P. (2017). Agricultural factors affecting *Fusarium* communities in wheat kernels. Int. J. Food Microbiol..

[60] Kuzdraliński A., Nowak M., Szczerba H., Dudziak K., Muszyńska M., Leśniowska-Nowak J. (2017). The composition of *Fusarium* species in wheat husks and grains in south-eastern Poland. J. Integr. Agric..

[61] Manstretta V., Rossi V. (2015). Effects of weather variables on ascospore discharge from *Fusarium graminearum* perithecia. PLoS ONE.

[62] ZMP—Zentrale Markt- und Preisberichtsstelle (2001). ZMP-Marktbilanz Getreide, Ölsaaten, Futtermittel 2001.

[63] Statistisches Bundesamt Landwirtschaftliche Bodennutzung. https://www.destatis.de/GPStatistik/servlets/MCRFileNodeServlet/DEHeft_derivate_00004307/2030312118004.pdf.

[64] Fernandez M.R., Chen Y. (2005). Pathogenicity of *Fusarium* species on different plant parts of spring wheat under controlled conditions. Plant Dis..

[65] Xu X.-M., Monger W., Ritieni A., Nicholson P. (2007). Effect of temperature and duration of wetness during initial infection periods on disease development, fungal biomass and mycotoxin concentrations on wheat inoculated with single, or combinations of, *Fusarium* species. Plant Pathol..

[66] Xu X., Nicholson P., Ritieni A. (2007). Effects of fungal interactions among *Fusarium* head blight pathogens on disease development and mycotoxin accumulation. Int. J. Food Microbiol..

[67] Lemmens M., Buerstmayr H., Krska R., Schuhmacher R., Grausgruber H., Ruckenbauer P. (2004). The effect of inoculation treatment and long-term application of moisture on *Fusarium* head blight symptoms and deoxynivalenol contamination in wheat grains. Eur. J. Plant Pathol..

[68] Lenc L., Czecholiński G., Wyczling D., Turów T., Kaźmierczak A. (2015). Fusarium head blight (FHB) and *Fusarium* spp. on grain of spring wheat cultivars grown in Poland. J. Plant Prot. Res..

[69] Rossi V., Ravanetti A., Pattori E., Giosuè S. (2001). Influence of temperature and humidity on the infection of wheat spikes by some fungi causing *Fusarium* head blight. J. Plant Pathol..

[70] Thrane U., Adler A., Clasen P.-E., Galvano F., Langseth W., Lew H., Logrieco A., Nielsen K.F., Ritieni A. (2004). Diversity in metabolite production by *Fusarium langsethiae*, *Fusarium poae*, and *Fusarium sporotrichioides*. Int. J. Food Microbiol..

