# Supplementary material for: Composition and Predominance of Fusarium Species Causing Fusarium Head Blight in Winter Wheat Grain Depending on Cultivar Susceptibility and Meteorological Factors
Source: Microorganisms. 2020 Apr 24;8(4):617. doi: 10.3390/microorganisms8040617 (PMC7232384; doi:10.3390/microorganisms8040617)
Supplement: Supplementary file 1 [file microorganisms-08-00617-s001.zip › Table S1 and S2.docx]

**Supplementary Material**

**Table S1.** Percentages of wheat grain samples infected with *F. graminearum* (*Fg*), *F. culmorum* (*Fc*), *F. avenaceum* (*Fa*), *F. poae* (*Fp*), *F. tricinctum* (*Ft*) and *F. langsethiae* (*Fl*) of the moderately to highly susceptible cultivar of the seven trial locations (three replications per location) in Northern Germany from 2013 to 2017 and across years. *n* = 105

| ***S*pecies** | **2013** | **2014** | **2015** | **2016** | **2017** | **2013-2017** |
| --- | --- | --- | --- | --- | --- | --- |
| ***Fg*** | 100 | 81 | 62 | 62 | 100 | 82 |
| ***Fc*** | 100 | 81 | 52 | 81 | 100 | 83 |
| ***Fa*** | 100 | 71 | 76 | 67 | 100 | 83 |
| ***Fp*** | 100 | 76 | 62 | 100 | 100 | 88 |
| ***Ft*** | 71 | 61 | 52 | 71 | 86 | 67 |
| ***Fl*** | 71 | 67 | 52 | 48 | 76 | 68 |

**Table S2.** Percentages of wheat grain samples infected with *F. graminearum* (*Fg*), *F. culmorum* (*Fc*), *F. avenaceum* (*Fa*), *F. poae* (*Fp*), *F. tricinctum* (*Ft*) and *F. langsethiae* (*Fl*) of the lowly to moderately susceptible cultivar of the seven trial locations (three replications per location) in Northern Germany from 2013 to 2017 and across years. *n* = 105

| ***S*pecies** | **2013** | **2014** | **2015** | **2016** | **2017** | **2013-2017** |
| --- | --- | --- | --- | --- | --- | --- |
| ***Fg*** | 100 | 71 | 52 | 57 | 100 | 76 |
| ***Fc*** | 100 | 71 | 52 | 76 | 100 | 80 |
| ***Fa*** | 100 | 67 | 61 | 67 | 100 | 79 |
| ***Fp*** | 100 | 81 | 57 | 91 | 100 | 86 |
| ***Ft*** | 57 | 43 | 38 | 57 | 81 | 55 |
| ***Fl*** | 67 | 52 | 48 | 43 | 57 | 53 |


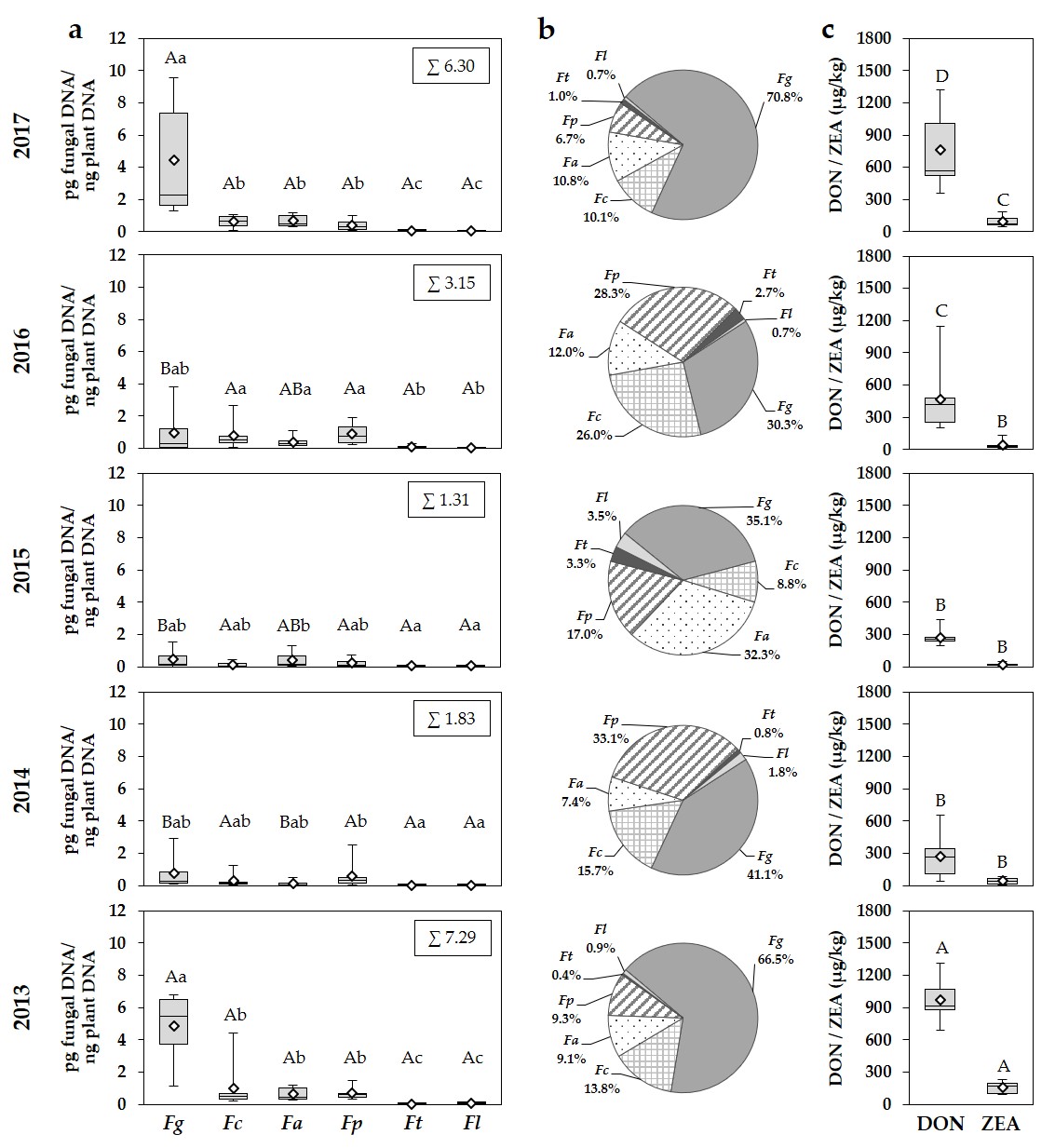


**Figure S1.** Boxplots and means (white rhombus) of (**a**) DNA amounts of *F. graminearum* (*Fg*), *F. culmorum* (*Fc*), *F. avenaceum* (*Fa*), *F. poae* (*Fp*), *F. tricinctum* (*Ft*) and *F. langsethiae* (*Fl*) (pg fungal DNA/ng plant DNA), (**b**) percentages of DNA amounts of the detected *Fusarium* species to the total *Fusarium* DNA amount of all detected species and boxplots and means (white rhombus) of (**c**) DON and ZEA concentrations (µg/kg) in wheat grain of the moderately to highly susceptible cultivar of the seven trial locations (three replications per location) in Northern Germany from 2013 to 2017. Five statistics are represented in each boxplot from bottom to top: the smallest observation, lower quartile, median, upper quartile, and largest observation, respectively. Mean values labelled with the same letter (small or big) are not significantly different from each other (*p* > 0.05). Capital letters describe differences for one species and DON and ZEA concentrations between years and small letters differences between the detected *Fusarium* species within a year. *n* = 105


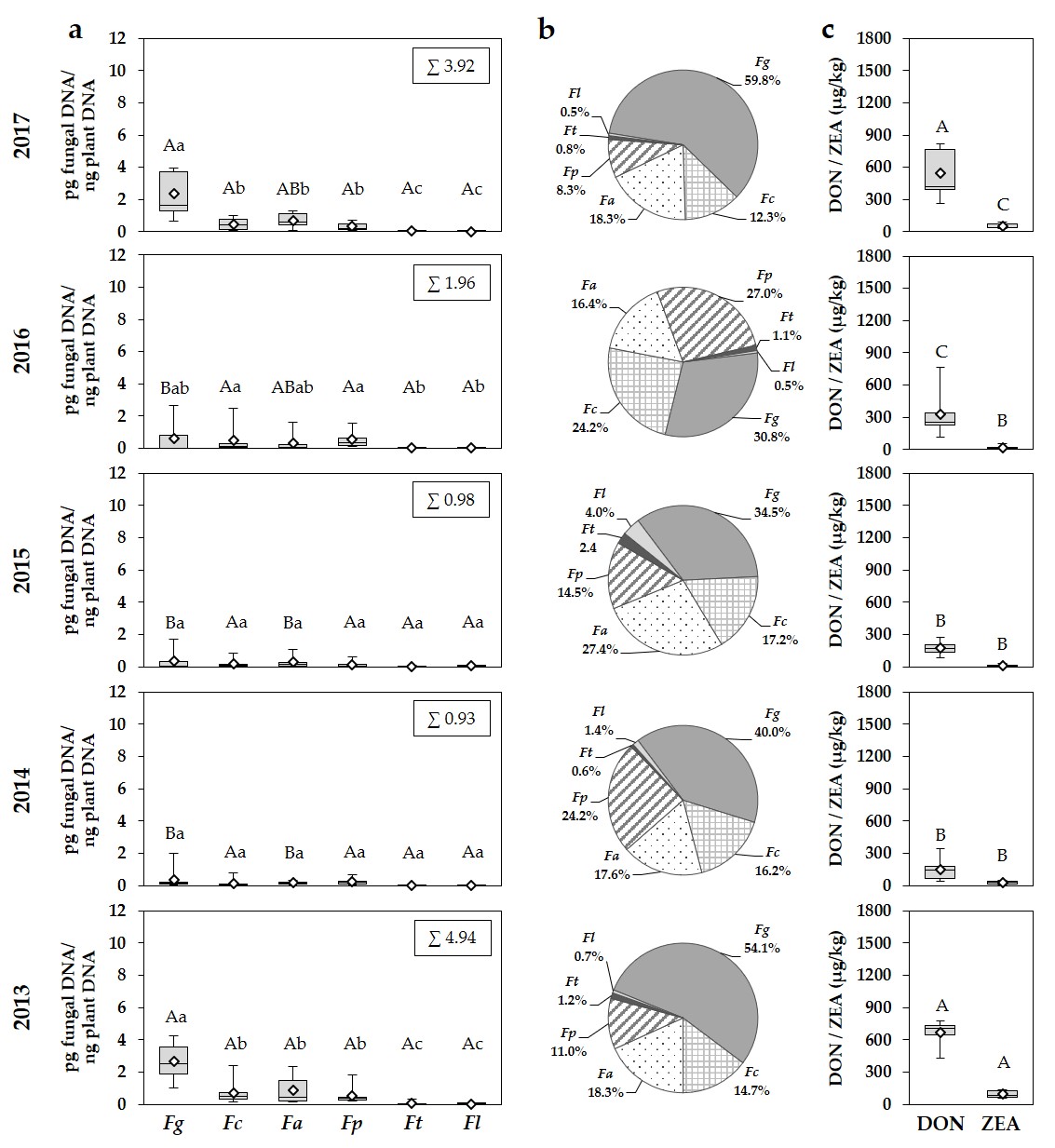


**Figure S2.** Boxplots and means (white rhombus) of (**a**) DNA amounts of *F. graminearum* (*Fg*), *F. culmorum* (*Fc*), *F. avenaceum* (*Fa*), *F. poae* (*Fp*), *F. tricinctum* (*Ft*) and *F. langsethiae* (*Fl*) (pg fungal DNA/ng plant DNA), (**b**) percentages of DNA amounts of the detected *Fusarium* species to the total *Fusarium* DNA amount of all detected species and boxplots and means (white rhombus) of (**c**) DON and ZEA concentrations (µg/kg) in wheat grain of the lowly to moderately susceptible cultivar of the seven trial locations (three replications per location) in Northern Germany from 2013 to 2017. Five statistics are represented in each boxplot from bottom to top: the smallest observation, lower quartile, median, upper quartile, and largest observation, respectively. Mean values labelled with the same letter (small or big) are not significantly different from each other (*p* > 0.05). Capital letters describe differences for one species and DON and ZEA concentrations between years and small letters differences between the detected *Fusarium* species within a year. *n* = 105
