# Supplementary figures and images for: Composition and Predominance of Fusarium Species Causing Fusarium Head Blight in Winter Wheat Grain Depending on Cultivar Susceptibility and Meteorological Factors
Source: Microorganisms. 2020 Apr 24;8(4):617. doi: 10.3390/microorganisms8040617 (PMC7232384; doi:10.3390/microorganisms8040617)

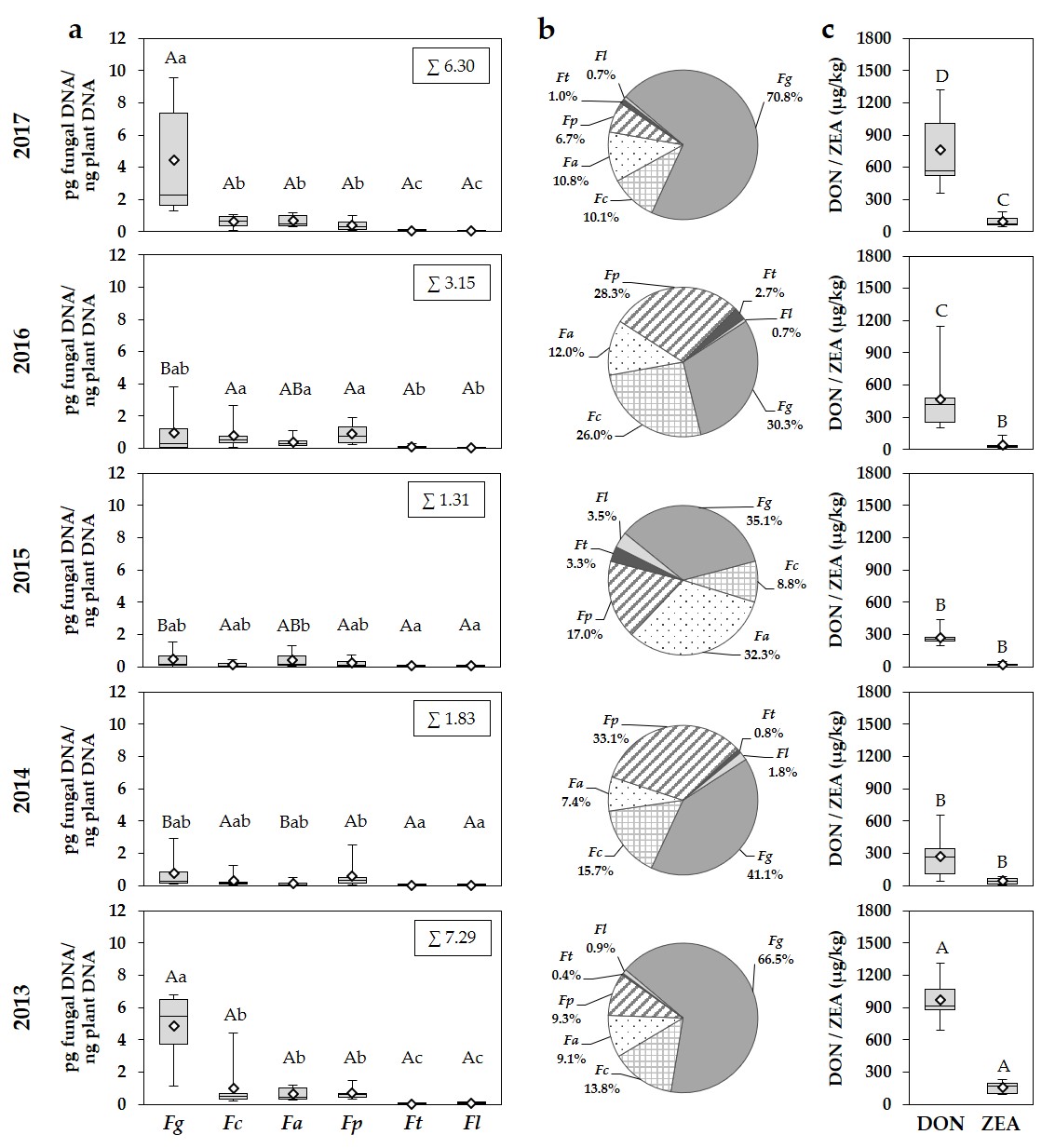

Supplement: Supplementary file 1 [file microorganisms-08-00617-s001.zip › Figure S1.jpg]

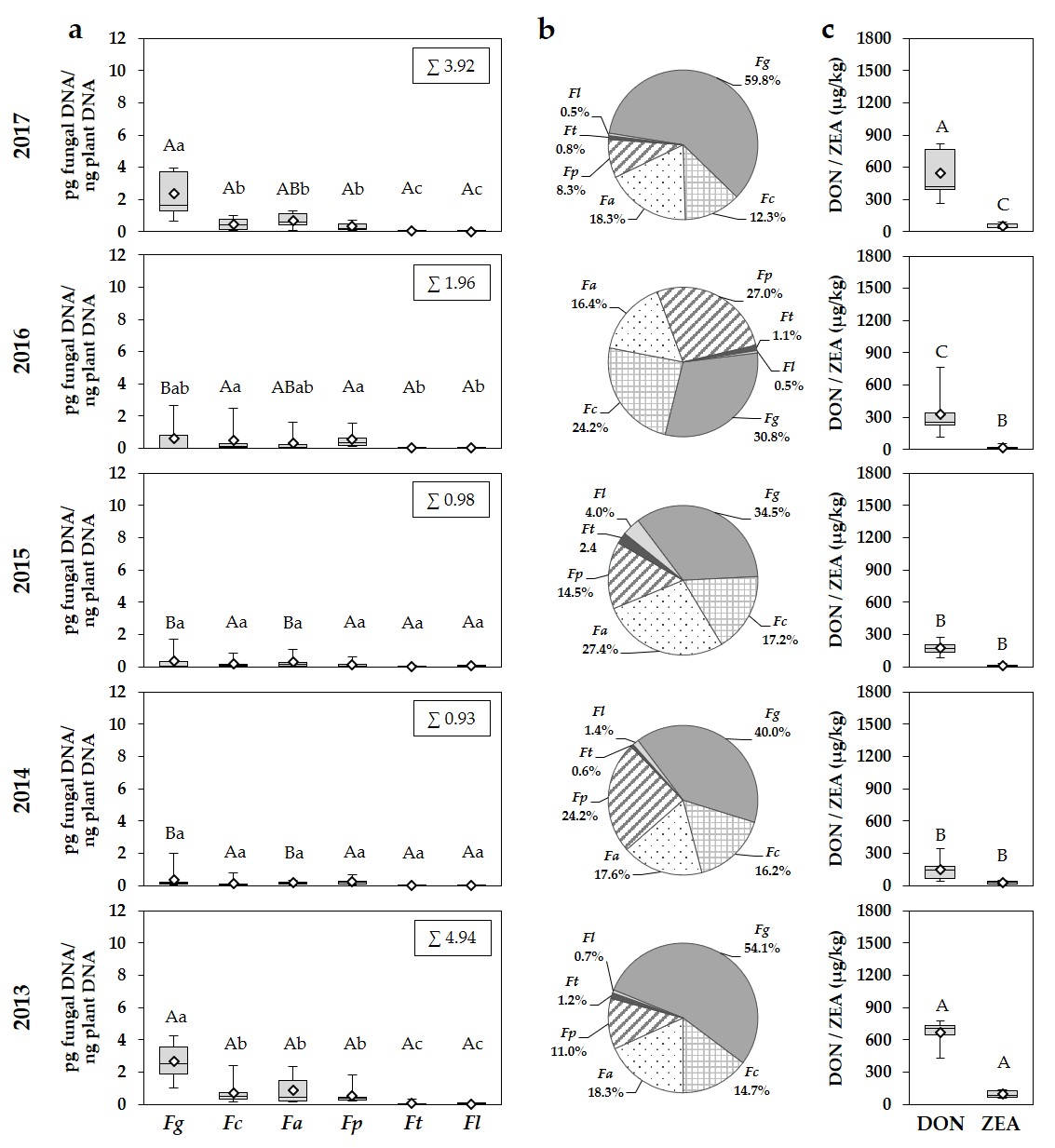

Supplement: Supplementary file 1 [file microorganisms-08-00617-s001.zip › Figure S2.jpg]
